# RhoA Ambivalently Controls Prominent Myofibroblast Characteritics by Involving Distinct Signaling Routes

**DOI:** 10.1371/journal.pone.0137519

**Published:** 2015-10-08

**Authors:** Aline Jatho, Svenja Hartmann, Naim Kittana, Felicitas Mügge, Christina M. Wuertz, Malte Tiburcy, Wolfram-Hubertus Zimmermann, Dörthe M. Katschinski, Susanne Lutz

**Affiliations:** 1 Institute of Pharmacology, University Medical Center Göttingen, Georg-August-University, Germany; 2 Department of Cardiovascular Physiology, University Medical Center Göttingen, Georg-August-University, Germany; 3 DZHK (German Center for Cardiovascular Research), partner site Göttingen, Germany; 4 Division of Cell and Molecular Biophysics, King´s College London, London, United Kingdom; University of Bergen, NORWAY

## Abstract

**Introduction:**

RhoA has been shown to be beneficial in cardiac disease models when overexpressed in cardiomyocytes, whereas its role in cardiac fibroblasts (CF) is still poorly understood. During cardiac remodeling CF undergo a transition towards a myofibroblast phenotype thereby showing an increased proliferation and migration rate. Both processes involve the remodeling of the cytoskeleton. Since RhoA is known to be a major regulator of the cytoskeleton, we analyzed its role in CF and its effect on myofibroblast characteristics in 2 D and 3D models.

**Results:**

Downregulation of RhoA was shown to strongly affect the actin cytoskeleton. It decreased the myofibroblast marker α-sm-actin, but increased certain fibrosis-associated factors like TGF-β and collagens. Also, the detailed analysis of CTGF expression demonstrated that the outcome of RhoA signaling strongly depends on the involved stimulus. Furthermore, we show that proliferation of myofibroblasts rely on RhoA and tubulin acetylation. In assays accessing three different types of migration, we demonstrate that RhoA/ROCK/Dia1 are important for 2D migration and the repression of RhoA and Dia1 signaling accelerates 3D migration. Finally, we show that a downregulation of RhoA in CF impacts the viscoelastic and contractile properties of engineered tissues.

**Conclusion:**

RhoA positively and negatively influences myofibroblast characteristics by differential signaling cascades and depending on environmental conditions. These include gene expression, migration and proliferation. Reduction of RhoA leads to an increased viscoelasticity and a decrease in contractile force in engineered cardiac tissue.

## Introduction

RhoA is a monomeric GTPase which is expressed in all cells and is activated by a plethora of upstream signaling cascades including important hormones and cytokines, RhoA controls fundamental cellular functions mainly via regulating the actin cytoskeleton [1–3]. In the cardiovascular system, the role of RhoA has been demonstrated in several cell types: In vascular smooth muscle cells RhoA controls the contractile function and thus regulates vascular resistance [4], in endothelial cells RhoA activation leads to barrier dysfunction [5], and in cardiomyocytes recent work demonstrates a protective role for RhoA in the scenario of ischemia/reperfusion [6]. In contrast to the increasing knowledge about the function of RhoA in many cardiovascular cell types, little is known about its role in cardiac fibroblasts.

Cardiac fibroblasts are a highly abundant cell type in the heart that under normal conditions control the homeostasis of the extracellular matrix (ECM) by producing matricellular proteins, matrix proteins as well as matrix degrading proteins [7]. In heart disease these cells become activated, start to produce increased amounts of ECM, proliferate and gain the ability to migrate. This overly activated phenotype of cardiac fibroblasts is called myofibroblast and is characterized by molecular changes including an increase in alpha-smooth muscle-actin (α-sm-actin) expression [8, 9]. Over time, the dysregulated cardiac fibroblast behavior leads to cardiac fibrosis which increases the stiffness of the heart muscle and impairs contractile function [10].

There is increasing effort to understand the molecular mechanisms driving the transition of cardiac fibroblasts to myofibroblasts. With respect to RhoA, so far mainly indirect findings document its involvement in processes leading to myofibroblast transition. Statins or other inhibitors which inhibit the essential isoprenylation of RhoA, have been shown to interfere with induced processes in myofibroblasts including cell proliferation [11–13]. Moreover, RhoA has recently been demonstrated to play a role in cardiac fibroblasts in the mineralcorticoid receptor-dependent regulation of the matricellular connective tissue growth factor (CTGF) [14]. CTGF is up-regulated in fibrotic heart disease and is thought to be involved in the regulation of ECM protein expression and in the control of angiogenesis and cardiomyocyte protection [15, 16]. In addition, the RhoA activating complex AKAP-Lbc was recently identified as a mediator of the angiotensin II-dependent RhoA activation in cardiac fibroblasts [17]. However, so far no detailed studies focusing on the role of RhoA and distinct downstream cascades and cellular processes in cardiac fibroblasts are available. Thus, we studied in detail the effects of a decrease in RhoA expression on cardiac (myo)fibroblast functions in 2D and 3D-cultures. Moreover, we unraveled the role of distinct downstream signal mediators in the relevant processes.

## Experimental Procedures

### Material

Primary and secondary antibodies were purchased from the following companies: *Primary antibodies*: acetylated tubulin (Sigma-Aldrich), α-actinin (Sigma-Aldrich), β-actin (Sigma-Aldrich), calsequestrin (Thermo Scientific), CTGF (Santa Cruz), Dia1 (Santa Cruz), H3 (Santa Cruz), HDAC6 (Cell Signaling), α-smooth-muscle actin (Sigma-Aldrich), RhoA (Santa Cruz), RhoC (Cell Signaling), tubulin (Sigma-Aldrich), tyrosinated tubulin (Sigma-Aldrich), vimentin (Sigma-Aldrich, Abcam), vinculin (Sigma-Aldrich), acetylated-lysine (Cell Signaling). *Secondary antibodies*: HRP-coupled antibodies (Sigma-Aldrich), Alexa Fluor 488-conjugated anti mouse (Jackson Immuno Research), Cy 3-conjugated anti mouse (Jackson Immuno Research), FITC-conjugated anti mouse IgG_1_ (SouthernBiotech), TRITC-conjugated anti mouse IgG_2b_ (SouthernBiotech).

For fluorescence labeling of actin filaments TRITC-phalloidin from Sigma-Aldrich and for indirect labeling of the Golgi apparatus AlexFluor488-wheat germ agglutinin (WGA) from Life Technologies was used.

The β- and γ-actin specific antibodies were a kind gift of Dr. Christine Chaponnier, Department of Pathology and Immunology, Geneva.

In experiments with wild type cardiac fibroblasts the following inhibitors were used: latrunculin A (LatA, 8.5 μM, Cayman), fasudil (10 μg/mL, LKT Laboratories), H1152p (300 nM, Cayman), tubastatin A (TubA, 5 μg/mL, Sigma-Aldrich)

*Primers*: PBGD (forw: cct gaa act ctg ctt cgc tg, rev: ctg gac cat ctt ctt gct gaa), HDAC6 (forw: agc gca gtc tta ttg atg gg, rev: cca tgc tca tag cgg tgg at), RhoA (forw: gca gat att gaa gtg gac ggg, rev: tgg gat gtt ttc taa act atc agg g), RhoB (forw: cat cga ctc gca caa agc ag, rev: ata cga tgc acg gag tgt cg), Biglycan (forw: ctg agg gaa ctt cac ttg ga, rev: cag ata gac aac ctg gag ga), Col1a (forw: acg cca tca agg tct act gc, rev: act cga acg gga atc cat cg), Col3a (forw: cca tga ctg tcc cac gta agc ac, rev: gga ggg cca tag ctg aac tga aaa c), α-sm-actin (forw: cat cag gaa cct cga gaa gc, tcg gat act tca ggg tca gg), TGF-β1 (forw: aga gcc ctg gat acc aac ta, rev: tgt tgg ttg tag agg gca ag).

### Methods

#### Ethics statement

Animal care and sacrifice was carried out in accordance with the guidelines (§4 Absatz 3 Deutsches Tierschutzgesetz) of the institutional animal care and use committee Niedersächsisches Landesamtes für Verbraucherschutz und Lebensmittelsicherheit (LAVES, Germany) which specifically approved this study (reference number T10/13). Rats were kept on a 12 h light/dark cycle with water and food ad libitum.

#### Cardiac fibroblast isolation

Cardiac fibroblasts were isolated from neonatal Wistar rats (day 0 to 3) by using a modified cell isolation protocol established by Webster et al. [18]. In brief, the animals were decapitated using strong surgical scissors and the atria of the isolated hearts were removed. Then ventricular tissues were cut in pieces and washed with CBFHH (0.14 M NaCl, 5.4 mM KCl, 0.81 mM MgSO_4_, 0.44 mM KHPO_4_, 0.34 mM Na_2_HPO_4_, 5.6 mM Glucose, 20 mM HEPES). Next, the tissue was dissociated by alternating incubation in trypsin and DNAseI solutions in CBFHH at room temperature while continuously shaking and gentle pipetting. The first supernatant was discarded, the following supernatents were collected and the digestion was stopped by adding fetal calf serum (FCS). After centrifugation for 15 min at 60 g and 4°C, the cells were pooled, washed with ice-cold DMEM containing 10% FCS and antibiotics and then filtered through a 250 μm mesh. The filtered cells were incubated for 60 min at 37°C, 5% CO_2_ on a cell culture plate at a density of 10^6^ cells per 145 cm^2^. Afterwards, the non-attached cardiomyocytes were removed and the attached cardiac fibroblasts were further cultivated in DMEM, 10% serum, 1% antibiotics, 1% non-essential amino acids until confluency was reached. Cells were only used from passage 0 to passage 2.

#### Lentivirus production and purification

For lentivirus production TsA201 cells were transfected with the pLKo.1-encoding shRNA plasmids (sense shRNA sequences: shRhoA: CCCAGACTAGATGTAGTATTT, shControl: CGGAATGACGAGCACACGAGA, scr: CCTAAGGTTAAGTCGCCCTCG), psPAX.2, PMD2.G and Polyfect according to the manufacturer’s protocol. After 48 and 72 h the virus particle containing supernatants were harvested, pooled and filtered through a 45 μm syringe filter to remove cell debris. The filtered supernatants were layered on a 30% sucrose cushion and centrifuged for 4 h at 22000 g and 4°C. The concentrated virus was collected, frozen in liquid nitrogen and stored at -80°C until further use.

#### Infection of cardiac fibroblasts with recombinant viruses

For lentiviral infection, the isolated cardiac fibroblasts were incubated with the purified lentiviruses in the presence of polybrene (8 μg/mL) in DMEM, 10% serum, 1% antibiotics, 1% non-essential amino acids and cultured afterwards for 48 h at 37°C and 5% CO_2_. Selection of the cardiac fibroblasts by puromycin (1 μg/mL) was started 4 to 5 days post infection and was performed up to 12 days. For adenoviral infection, the lentiviral transduced or wild type cardiac fibroblasts were incubated with the LifeAct adenovirus (Life Technologies) for 24 h and then directly used for migration assays.

#### siRNA tranfection

For siRNA transfection the NRCF were seeded at a density of 70% in DMEM, 10% serum, 1% antibiotics, 1% non-essential amino acids. Transfection was performed using Lipofectamin 2000 (Life Technologies) according to manufacturer´s protocol in Opti-MEM (Life Technologies). After 6–8 h the transfection solution was changed into culture medium and the cells were incubated for 48 h. *siRNA sequences*: siControl (uucuccgaacgugucacgu), siDia1 (gcgacggcggcaaacauaagaaauu).

#### Immunoblot analyses

For analysis of extracellular CTGF medium samples were collected from the cell culture dish prior to cell lysis. To prepare the lysate and the particulate fraction the cells were washed once with cold PBS and then scraped off in a buffer containing 50 mM Tris pH 7.4, 150 mM NaCl, 4 mM MgCl_2_, 10% Glycerol (v/v), 1% Igepal CA–630 (v/v). After centrifugation at 15000 g for 10 min at 4°C, the particulate fractions were treated with ultrasound in SDS-loading buffer to reduce sample viscosity. All samples were used for SDS-PAGE and subsequent blotting. The membranes were incubated with Rotiblock for 1 h, incubated with the primary antibodies over night at 4°C and the HRP-coupled-secondary antibodies for 1 h at room temperature. A chemoluminescence reaction was performed and the signals documented and analyzed with the Versadoc system (Biorad).

#### Rho activity binding assay

The transduced and selected fibroblasts were kept in low serum conditions for 24 h. Then, the cells were kept on a heating plate at 37°C and stimulated with 100 nM Angiotensin II for 30 seconds. The cells were lysed with ice-cold GST-Fish buffer (10% Glycerol, 50 mM Tris pH 7.4, 100 mM NaCl, 2 mM MgCl_2_, 1% Igepal) and the debris was pelletized by centrifugation. To determine total RhoA expression 50 μL of the supernatants were stored separately on ice. The remaining lysate was incubated with the GST-Rho-binding domain of rhotekin (RBD)-fusion protein bound to glutathione sepharose which exclusively binds the active forms of RhoA, B and C. The mixture was incubated for 1 h on ice in a rotating shaker. The sepharose was separated from the lysates by centrifugation. The supernatant was removed and the sepharose washed twice with GST-Fish buffer. The samples were incubated up in SDS-loading buffer at 95°C for 5 min. Analysis of the total and the activated amount of RhoA was performed by immunoblot analyses.

#### Quantitative PCR

Total RNA from transduced and selected fibroblasts was isolated using the RNeasy kit (Qiagen) and first strand cDNA was synthesized using the RevertAid first strand cDNA synthesis kit (Thermo Scientific). The cDNA was diluted 1:20 and quantitative PCR was performed using the Taqman 7900 HT (Life Technologies) and SYBR Green reagents (Qiagen). Samples were run in quadruplicates each using the primers listed above. The data was calculated using a standard curve and normalized to PBGD as housekeeping gene.

#### Immunofluorescence and f-actin staining

The cells were washed with cold PBS, fixed for 15 min in 4% paraformaldehyde in PBS, washed with PBS and permeabilized with 0.05% Triton X–100 in PBS for 3 min. Blocking was performed using Immunoblock for 1 h at room temperature, and cells were incubated with the respective primary antibodies over night at 4°C. After one washing step, the fluorophore-coupled secondary antibodies were incubated together with DAPI for 1h at room temperature in the dark and finally the cells were washed once with PBS. Alternatively, the cells were incubated with 0.5 μg/mL TRITC-labeled phalloidin, 5 μg/mL Alexa Fluor 488-labeled WGA and 1 μg/mL DAPI after blocking for 1 h and then washed with PBS.

#### Microscopy and morphometric analyses

The fluorescent stainings were analyzed using an inverted fluorescence microscope (Olympus) with the following specifications: microscope IX–81 inverted fluorescence microscope (Olympus), camera: XM10 (Olympus), objectives: UPlanFLN4xPh, UPlanFLN10xPh, LUCPlanFLN20xPh, LUCPlanFLN40xPh, filters: 350 DAPI, 575 TxRed, 485 FITC, climate chamber: Incubator OL IX81 2000, software Xcellence pro (Olympus). All morphometric analyses were performed with the software Image J (Version 1.49o).

#### Adhesion assay

The transduced and selected cells were seeded in DMEM, 10% serum, 1% antibiotics, 1% non-essential amino acids with a density of 100 cells per cm^2^ in a 12-well plate with and without collagen coating. After 15, 30, 45 and 60 min brightfield images were taken randomly. The adherent and non-adherent, round cells were counted and the percentage of each fraction was calculated.

#### Analysis of cell viability

Transduced and selected cells were seeded in 12-well plates, after reaching 80% confluency washed with PBS and then incubated with a dye mixture (Roche) containing annexin V-FITC and propidium iodide for 15 min at room temperature. The stainings were visualized with an inverted fluorescence microscope and the numbers of green (apoptotic) and green/red (necrotic) cells were evaluated and given as percentages of the total cell number.

#### Analysis of cell migration

**Migration on a focal plane:** Transduced and selected or wild type cardiac fibroblasts were seeded in DMEM, 10% serum, 1% antibiotics, 1% non-essential amino acids in a density of 100 cells per cm^2^ on a 24-well plate. The wild type cells were treated with the respective inhibitor. The cells were transduced using the LifeAct^®^-GFP adenovirus and incubated for 48 hours. Live cell imaging was performed in the IX–81 inside the climate chamber at 37°C, 5% CO_2_ for 24 hours. An image was taken every 20 min and the cells were tracked using the manual tracking plug-in for Image J. Total distance, average velocity and directness of migrating cells was calculated using the chemotaxis and migration tool provided by Ibidi.

**Transwell migration:** Transwell migrationFor the investigation of transwell migration, thincerts (Greiner Bio One) with a pore size of 8 μm were used. The thincerts were inserted into a 24-well plate and filled with DMEM, 1% antibiotics, 1% non-essential amino acids and either 1% or 10% serum. Per cavity 5000 transduced and selected cardiac fibroblasts or wild type cardiac fibroblasts treated with the respective inhibitors were seeded and incubated for 24 hours. The thincerts were then washed with PBS and the cells fixed for 15 min in 4% paraformaldehyde in PBS. The membranes were removed, placed on a microscope slide and incubated with Hoechst33342 (10 μm/mL) for 30 min. For evaluation the cells found in the pores of the membrane were visualized by fluorescence microscopy and counted.

**Migration through a collagen matrix:** The channel of a μ^3D^-microscope slide provided by ibidi was filled with a collagen matrix (2% w/v, 10xDMEM in water, pH 7.4) and incubated at 37°C for 30 minutes. Transduced NRCF were seeded confluently in low serum condition in the left chamber while the right chamber was filled with high serum medium. After 24 h cells in the chambers were fixed and stained with TRITC-labeled phalloidin and DAPI as described and the area inside the collagen matrix, which was covered by cells, was quantified.

#### Analysis of cell proliferation

The transduced and selected or wild type cardiac fibroblasts were seeded in a density of 5000 cells per well in a 96-well plate and incubated for 1–4 days at 37°C and 5% CO_2_ in the presence of 1% or 10% FCS in DMEM. The wild type cells were additionally treated with fasudil, H1152p or tubastatin A (TubA) as indicated. The medium with the respective supplements was changed daily. At each time point, the cells were fixed for 15 min in 4% paraformaldehyde in PBS, washed with PBS and permeabilized with 0.05% Triton X–100 in PBS for 3 min. After incubation with 1 μg/mL DAPI for 30 min at room temperature the assay was evaluated using the Cellavista System (Roche).

#### Serum response factor activation—Luciferase Assay

The transduced and selected cardiac fibroblasts were seeded at a density of 50.000 cells/well in a 24 well plate and maintained in high or low serum medium (DMEM, 10% or 0% serum, 1% antibiotics, 1% non-essential amino acids) for 2 days. Transfection of pSRE.L and pRL.TK plasmids was conducted using the Lipofectamin 3000 kit (Life Technologies) according to manufacturer´s protocol. Luciferase activities were determined using the Dual-Glo luciferase assay system and the Glomax luminometer (both Promega). The firefly luciferase values were normalized to renilla luciferase.

#### EHM—engineered heart muscle

A suspension of 2*10^6^ isolated cardiac cells was complemented with 0.5*10^6^ of transduced and selected NRCF and mixed with collagen type I (rat tail), a basement membrane protein mixture (Matrigel, BD) and concentrated serum-containing culture medium (2xDMEM in water, supplemented with 20% horse serum, 4% chick embryo extract (CEE) and 1% antibiotics, pH adjusted to 7.4 with NaOH). The mixture was casted into circular molds and incubated at 37°C and 5% CO_2_, after 1 h DMEM, 10% horse serum, 2% CEE and 1% antibiotics was added. The medium was changed after 24 h and later every two days. After 7 days of culture the condensed EHM were transferred on phasic stretchers and stretched at 1 Hz for 24 h, then at 2 Hz for another 6 days while medium change was continued as described before. Following phasic stretch the EHM were transferred to an organ bath at 37°C containing Tyrode´s solution (119.8 mM NaCl, 5.4 mM KCl, 0.2 mM CaCl_2_, 1.05 mM MgCl_2_, 0.42 mM NaH_2_PO_4_, 22.6 mM NaHCO_3_, 5 mM glucose, 0.28 mM ascorbic acid) and continuously gassed with 95%/5% O_2_/CO_2_. The rings were paced at 2 Hz with a calcium concentration of 1.8 mM and stretched untill L_max_, the length at which the maximal force is generated, was reached. After buffer change and equilibration the contraction force of the tissues with increasing Ca^2+^ concentration from 0.2 mM to 3.2 mM was measured.

#### Protein extraction from EHM

EHM were crushed with a pestle in a liquid nitrogen-cooled metal block and transferred into lysis buffer containing 50 mM Tris pH 7.4, 150 mM NaCl, 4 mM MgCl_2_, 10% Glycerol (v/v) and 1% Igepal CA–630 (v/v). After incubation on ice for 15 min the samples were mixed, centrifuged and the lysate separated from the particulate for further analysis by immunoblot.

#### Engineered connective tissue (ECT)

A suspension of 1.7 *10^6^ lentivirally transduced and selected NRCF were casted with a matrix of collagen (rat tail) and concentrated serum-containing culture medium (2.5xDMEM in water, supplemented with 1% antibiotics, pH adjusted to 7.4 with NaOH) into circular molds. After condensation (37°C, 5% CO2, 1 h) fresh culture medium (DMEM, 10% serum, 1% antibiotics, 1% non-essential amino acids) was added and changed every second day. At day 7 the size of the ECT was determined, rheometric destructive tensile strength measurements were performed (RSA-G2, TA Instruments) and the Young´s modul and failing points were determined.

#### Coculture experiments

Freshly isolated neonatal rat cardiomyocytes (NRCM) were cultivated (DMEM, 10% serum, 1% antibiotics 0.5% BrdU) for 4 days. Lentivirally transduced and selected NRCF were seeded ton top of the NRCM population. The co-culture was maintained for 7 days, cells were lysed and prepared for immunoblot analysis as described.

#### Statistical Analysis

Results are presented as means ± SEM. Data was analyzed by 1way ANOVA, followed by Bonferroni's Multiple Comparison Test or by unpaired t-test. p values of less than 0.05 were considered as statistically significant and are indicated.

## Results

### RhoA controls the cytoskeleton in NRCF

**Fig 1:**
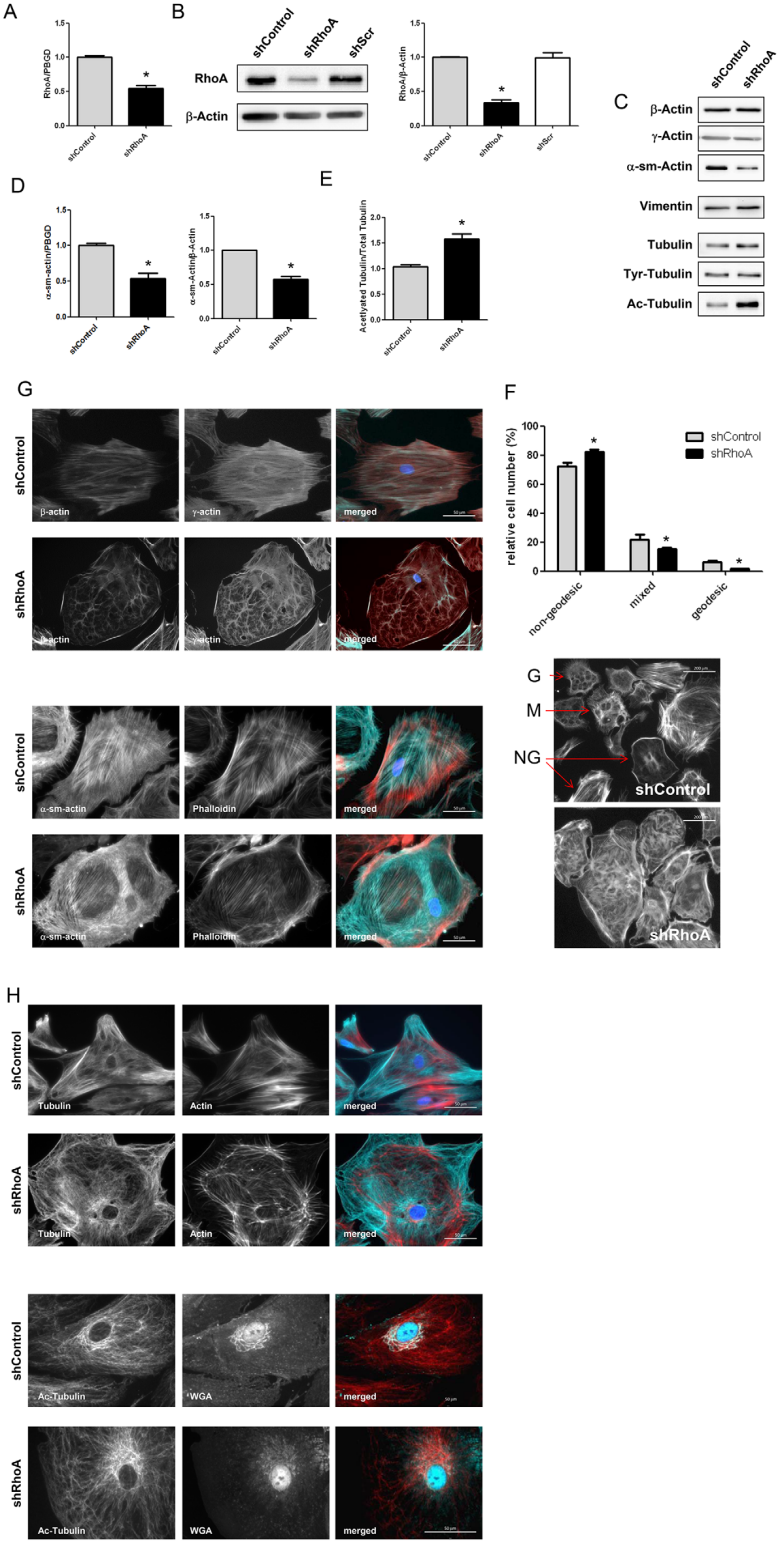
RhoA knockdown influences cytoskeletal protein expression and localization as well as disturbs higher order actin structures. A) Bar graph summary of real-time PCR data for RhoA in shControl and shRhoA NRCF normalized to PBGD and related to shControl (means ± SEM, n = 6 versus PBGD, *p < 0.05). B) Representative immunoblot of RhoA and β-actin in whole cell lysates from shControl, shScr and shRhoA NRCF (left). Bar graph summary of RhoA protein expression normalized to β-actin (right). The relative change of RhoA in shRhoA NRCF and shScr NRCF to shControl NRCF is given (means ± SEM, n = 22, *p < 0.05). C) Representative immunoblots of investigated cytoskeletal proteins in whole cell lysates from shControl and shRhoA NRCF are shown. D) Bar graph summary of real-time PCR data of α-sm-actin normalized to PBGD and protein expression normalized to β-actin in shControl and shRhoA NRCF relative to shControl (means ± SEM, n = 7, *p < 0.05). E) Bar graph summary of acetylated tubulin normalized to β-actin. Whole cell lysates obtained from shControl and shRhoA NRCF were used. The values are given relative to shControl (means ± SEM, n = 7–12, *p < 0.05). F) Immunofluorescence staining of shControl and shRhoA NRCF for actin isoforms β- (green), γ-actin (red) and DAPI (blue) (320x, upper two rows) or for α-sm-actin (green), actin (red) and DAPI (blue) (lower two rows). G) Analysis of geodesic actin structures (left) and representative immunofluorescence staining of actin structures in shControl and shRhoA NRCF (right). The relative number of geodesic (G), mixed (M) and non-geodesic (NG) NRCF as indicated (arrow) in shControl compared to shRhoA is given (means ± SEM, n = 3, *p < 0.05). H) Immunofluorescence staining of tubulin (green), actin (red) and DAPI (blue) (400x, upper two rows) or acetylated tubulin (red), Golgi apparatus (green) and DAPI (blue) (400x, lower two rows) in shControl and shRhoA NRCF.

In order to study the role of RhoA in primary cardiac fibroblasts we used a lentiviral shRNA approach and achieved a mean knockdown of about 50% on mRNA and 60% on protein level (Fig 1A and 1B). We ensured that the experimental procedure does not affect RhoA activation in shControl cells but inhibits it in shRhoA NRCF (S1 Fig). In addition, we verified that the expression of the two homologs RhoB and RhoC were not affected in shRhoA cells (S1 Fig). We then analyzed the expression of several signal mediators (data not shown) and cytoskeletal proteins (Fig 1C) and found that the myofibroblast marker α-sm-actin was decreased in shRhoA NRCF by 50% on RNA and protein level (Fig 1D). Tubulin acetylation was increased by 50% (Fig 1E), however, no changes in the tubulin deacetylase HDAC6 expression was found (S1 Fig). Moreover the expression of β-actin, γ-actin, vimentin and tubulin was changed (Fig 1C). To further analyze whether other proteins than tubulin are differently acetylated we performed immunoblot analyses from cell lysates with an anti-acetylated-lysine antibody but could not detect other changes (S1 Fig).

In the next step, the organization of the cytoskeleton was studied using fluorescence analyses. Staining of β-actin, γ±-actin, α-sm-actin as well as of total actin with phalloidin showed a less organized actin network in shRhoA compared to shControl NRCF (Fig 1F). Quantification of higher order actin structures, including geodesic domes and partially geodesic variants revealed that less of these structures could be detected in shRhoA NRCF, and instead internal star-shaped actin bundles and cortical actin structures were present (Fig 1G). With respect to tubulin and its posttranslational modification we could show that in shRhoA cells the acetylated tubulin which can be preferentially detected in the perinuclear region and in zones where the Golgi apparatus resides was increased (Fig 1H).

### RhoA regulates the Golgi apparatus compactness

**Fig 2:**
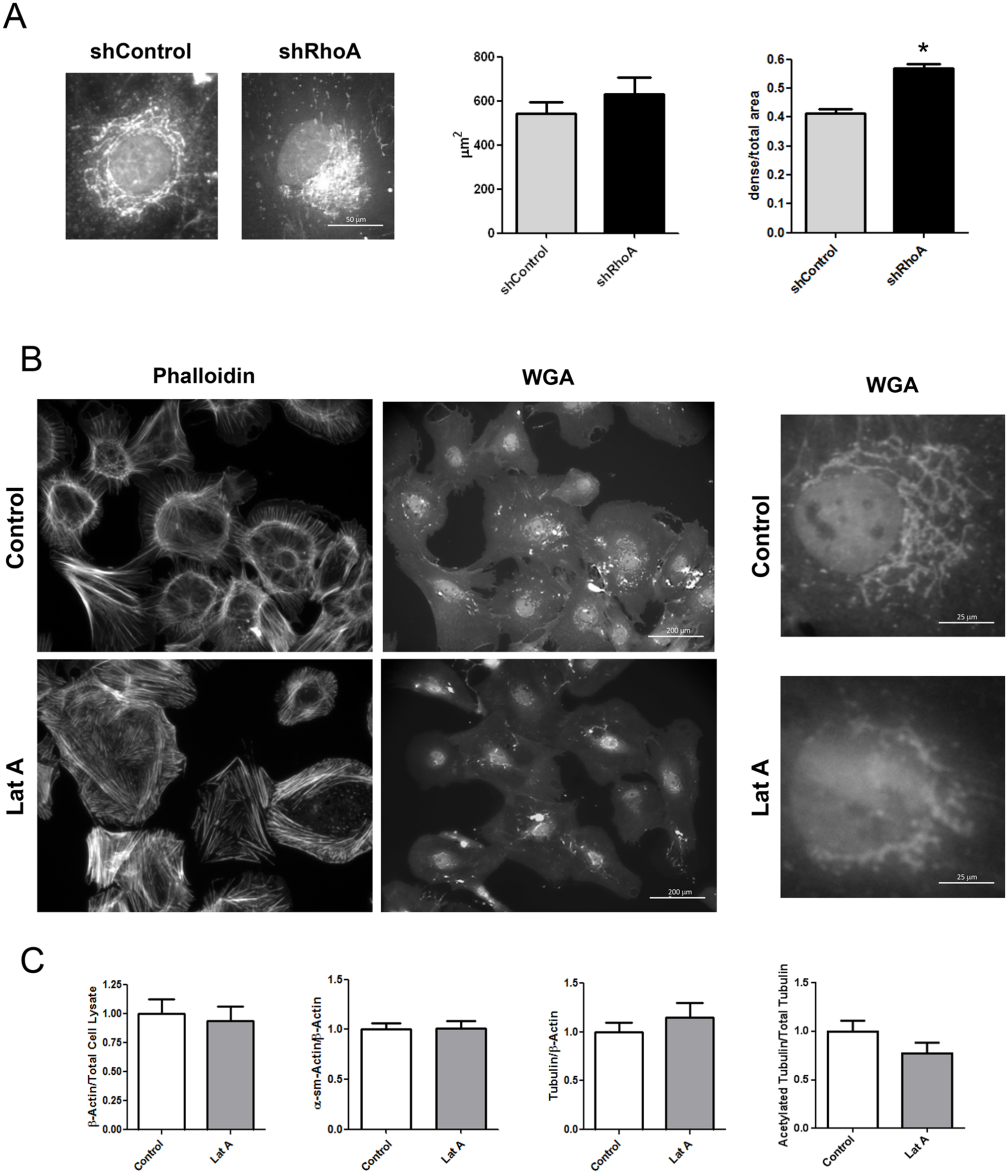
Knockdown of RhoA and actin depolymerization by Latrunculin A (LatA) results in changes in the Golgi apparatus structure. A) Representative immunofluorescence staining of the Golgi apparatus (640x, left) and structural analysis of Golgi apparatus size and density (right) of shControl and shRhoA NRCF (means ± SEM, n = 30, *p < 0.05). B) Immunofluorescence staining of actin (left) and Golgi apparatus (middle) in control and LatA treated NRCF (8.5 μM, 200x) In addition, higher magnifications are shown (640x). C) Bar graph summary of immunoblot analysis of β-actin normalized to total cell lysate protein, α-sm-actin, tubulin normalized to β-actin and acetylated tubulin normalized to total tubulin. Whole cell lysates were obtained from control and LatA treated NRCF. The relative change of expression in LatA treated NRCF to control NRCF is given (8.5 μM, n = 7).

Next, the morphology of the Golgi apparatus was analyzed by quantification of the area and compactness of WGA-positive membranes. No changes were found in Golgi area but the density of Golgi membrane stacks was higher in shRhoA NRCF (Fig 2A). To analyze whether this is due to the disturbed actin cytoskeleton or the increase in tubulin acetylation we incubated wild type cardiac fibroblasts with the actin depolymerizing agent latrunculin A (LatA) and studied Golgi apparatus morphology as well as tubulin expression and acetylation. In the used concentration LatA only partly destabilized the actin cytoskeleton similar to the knockdown of RhoA and led to an increase in Golgi compactness (Fig 2 Golgi) as already described before [19], but did not change the expression and modification of tubulin. LatA treatment had also no effect on the expression of actin isoforms (Fig 2C).

### RhoA regulates NRCF adhesion and polarity

**Fig 3:**
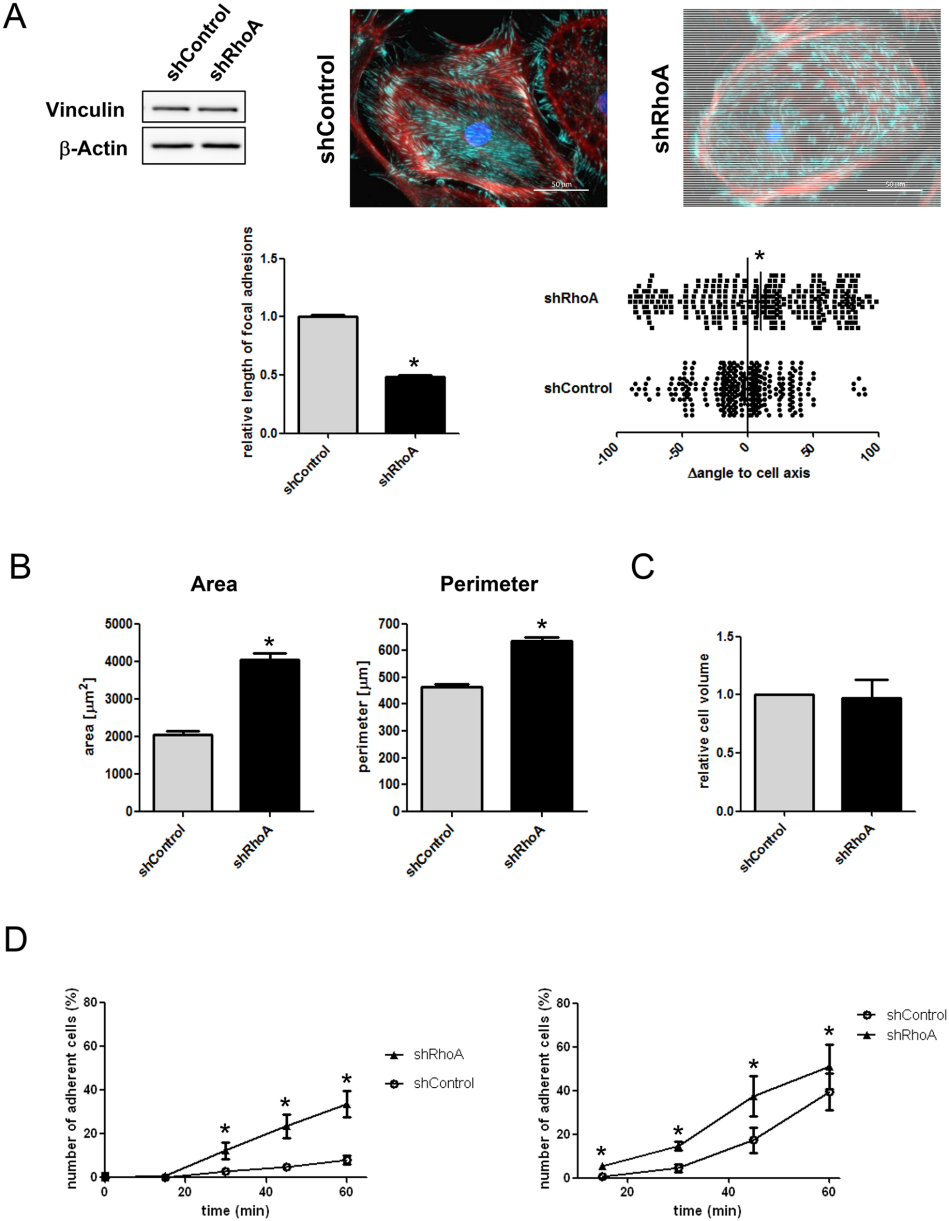
RhoA knockdown changes focal adhesion site size and orientation as well as cell morphology and adhesion velocity. A) Representative immunoblot of vinculin and β-actin in whole cell lysates from shControl and shRhoA NRCF (upper left). Immunofluorescence stainings of vinculin (green), actin (red) and DAPI (blue) in shControl and shRhoA NRCF is shown (upper right) (320x). Analysis of focal adhesion site length (lower left) and orientation to the cell axis (lower right) in shControl and shRhoA NRCF is depicted (means ± SEM, n = 3, at least 110 focal adhesion sites per condition were analyzed, *p < 0.05). B) Morphometric analysis of cell area (left) and perimeter (right) of shControl and shRhoA NRCF (means ± SEM, n = 3, each 50 NRCF per condition, *p < 0.05). C) Cell volume of detached shControl and shRhoA NRCF measured by resistance in a pulsed low voltage field. Given is the relative cell volume of shRhoA compared to shControl NRCF (n = 3) D) Adhesion assay on cell culture (left graph) and collagen-coated surface (right graph) over a time course of 1 h. The number of adherent shRhoA compared to shControl NRCF in percent of total cell number is given for each time point (means ± SEM, n = 4, *p < 0.05).

The disturbance of the actin cytoskeleton in shRhoA cells is also reflected by a change in morphology and orientation of focal adhesion sites. The adhesion sites, as assessed by vinculin staining, were significantly smaller and most importantly the predominant arrangement along the cell axis was disturbed, arguing for a loss in cell polarity (Fig 3A). Morphometric analyses revealed that shRhoA NRCF, when attached display an increased surface and perimeter, without showing an increased cell volume when detached (Fig 3B and 3C). In adhesion assays we found that the initial adhesion phase is faster in shRhoA cells independent of the substratum (Fig 3D).

### RhoA regulates NRCF migration

**Fig 4:**
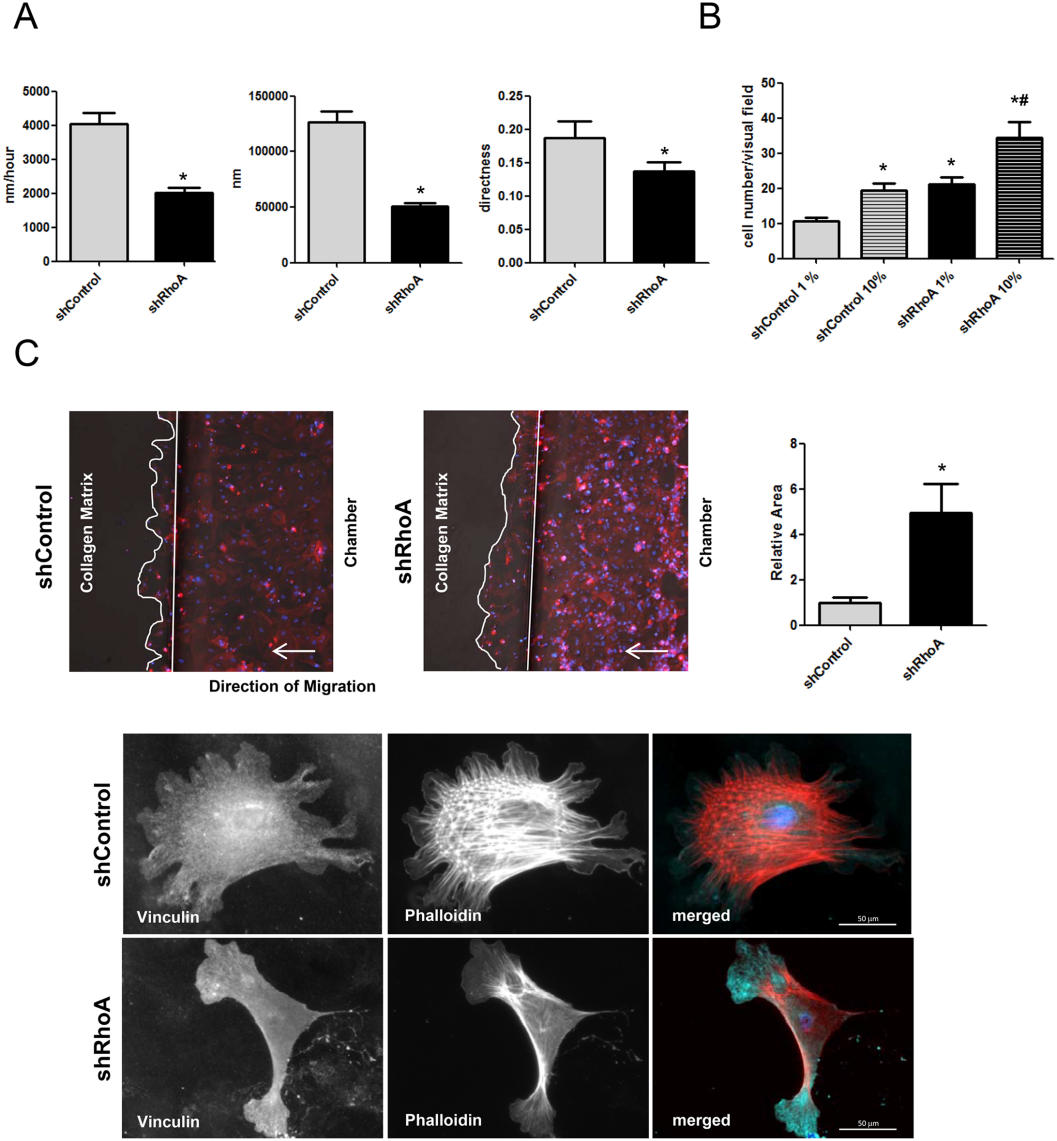
Downregulation of RhoA inhibits migration on a focal plane but improves amoeboid migration performance. A) Bar graph summary of absolute distance (left), average migration velocity (middle) and directness (right) of shControl and shRhoA NRCF migrating on a plane cell culture surface (means ± SEM, n = 3, 15 NRCF per virus type, *p < 0.05). B) Bar graph summary of cell migration of shControl and shRhoA NRCF through a porous membrane (pore size: 8 μm) in the presence of low serum (1%) and high serum (10%) (means ± SEM, n = 5, *p < 0.05). C) Representative images of shControl and shRhoA cells migrating through a collagen membrane in ibidi μ slides^3D^. The cells were stained for total actin and with DAPI (left). Bar graph summary of cell migration of shControl and shRhoA NRCF through a collagen matrix (1,5% in DMEM) in ibidi μ-slides^3D^. As a bait FCS (10%) was used (means ± SEM, n = 8, *p < 0.05) (right). Immunofluorescence staining of vinculin (green), actin (red) and DAPI (blue) (320x) of shControl (bottom, upper panel) and shRhoA NRCF (bottom, lower panel) inside a collagen matrix (1,5% in DMEM).

To evaluate whether the observed morphological changes impact the migratory behavior of shRhoA NRCF we studied migration on a plane surface. Although, the migration capacity of these cells is relatively low compared to other cell types, our data shows that on a plane surface the knockdown of RhoA led to a decrease in migration velocity, a reduced migration distance and a lower directness of migration (Fig 4A, S1 and S2 Videos). On the other hand, in a transwell assay in which the capacity of the shRhoA NRCF to migrate through pores was studied, an increase in cell migration was found independent of the used serum concentrations in the chemoattracting medium (Fig 4B). To asses this unexpected finding further, we used an *in matrix*-migration approach in which the entrance into and migration through a collagen-containing chamber was analyzed. Similar to the transwell assay, the shRhoA NRCF showed a higher migratory response in this assay (Fig 4C). F-actin staining demonstrates that in comparison to control cells, shRhoA NRCF show less internal actin fibers and more pronounced protrusions at the migratory front of the cell (Fig 4C).

### RhoA regulates NRCF proliferation

**Fig 5:**
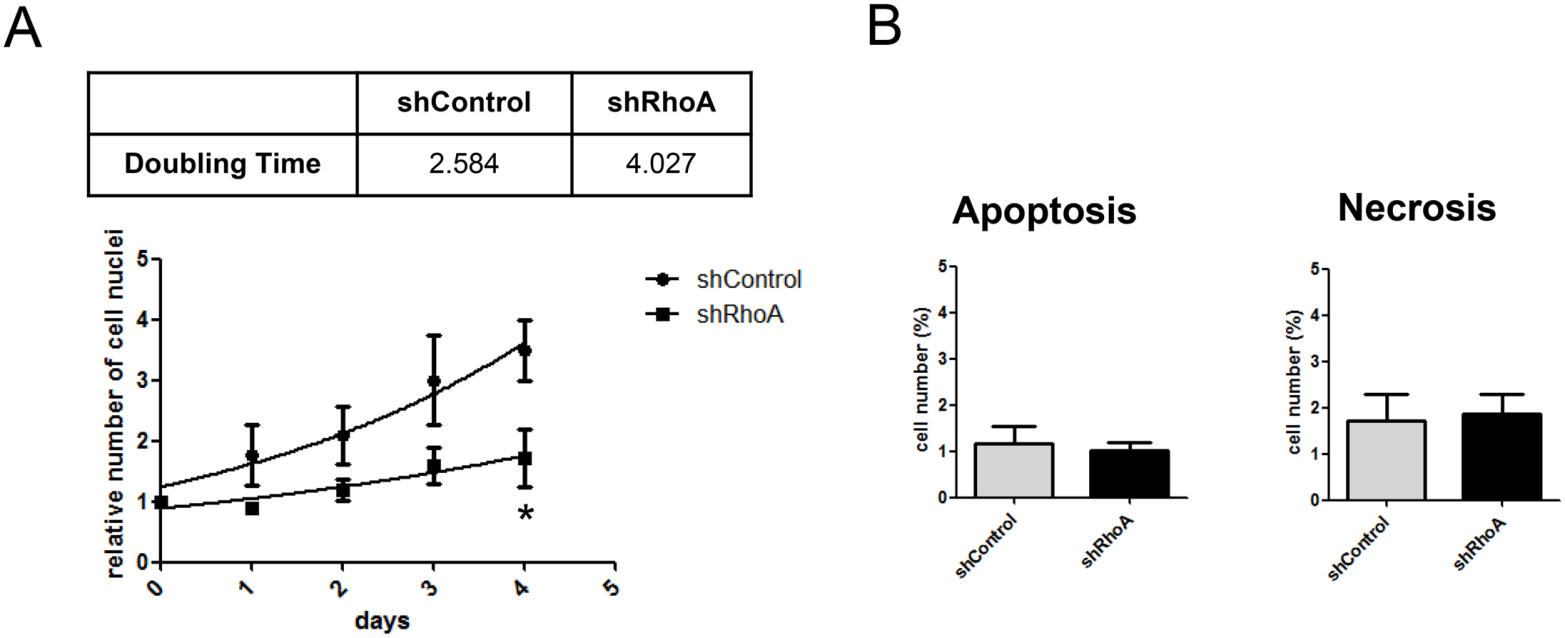
The knockdown or RhoA reduces cell doubling time without affecting the cell viability. A) Proliferation-DAPI assay of shControl and shRhoA NRCF over a time course of 4 days. Doubling time of the involved cell types is shown in the table above (doubling time in days) (means ± SEM, n = 5, measured in 8 replicates each, *p < 0.05). B) AnnexinV-FLUOS and propidium iodide staining of shControl and shRhoA NRCF. Bar graph summary of apototic (left) and necrotic (right) NRCF in percent compared to total cell number (means ± SEM, n = 3).

Another characteristic feature of myofibroblasts is their increased proliferation capacity compared to quiescent fibroblasts. Thus, we studied NRCF proliferation in the presence of serum. By reducing the expression of RhoA the serum-driven proliferation was strongly impaired with 2.6 and 4.0 days of doubling time for shControl and shRhoA NRCF, respectively (Fig 5A). In the absence of serum the overall proliferation capacity of all cells analyzed was extremely low (data not shown). By performing viability measurements using a combined staining with annexin V and propidium iodide we found no differences in cell viability (Fig 5B). Therefore, an apoptotic or necrotic process can be excluded as a cause for the decreased proliferation capacity of shRhoA NRCF.

### RhoA influences expression of secreted proteins in NRCF

**Fig 6:**
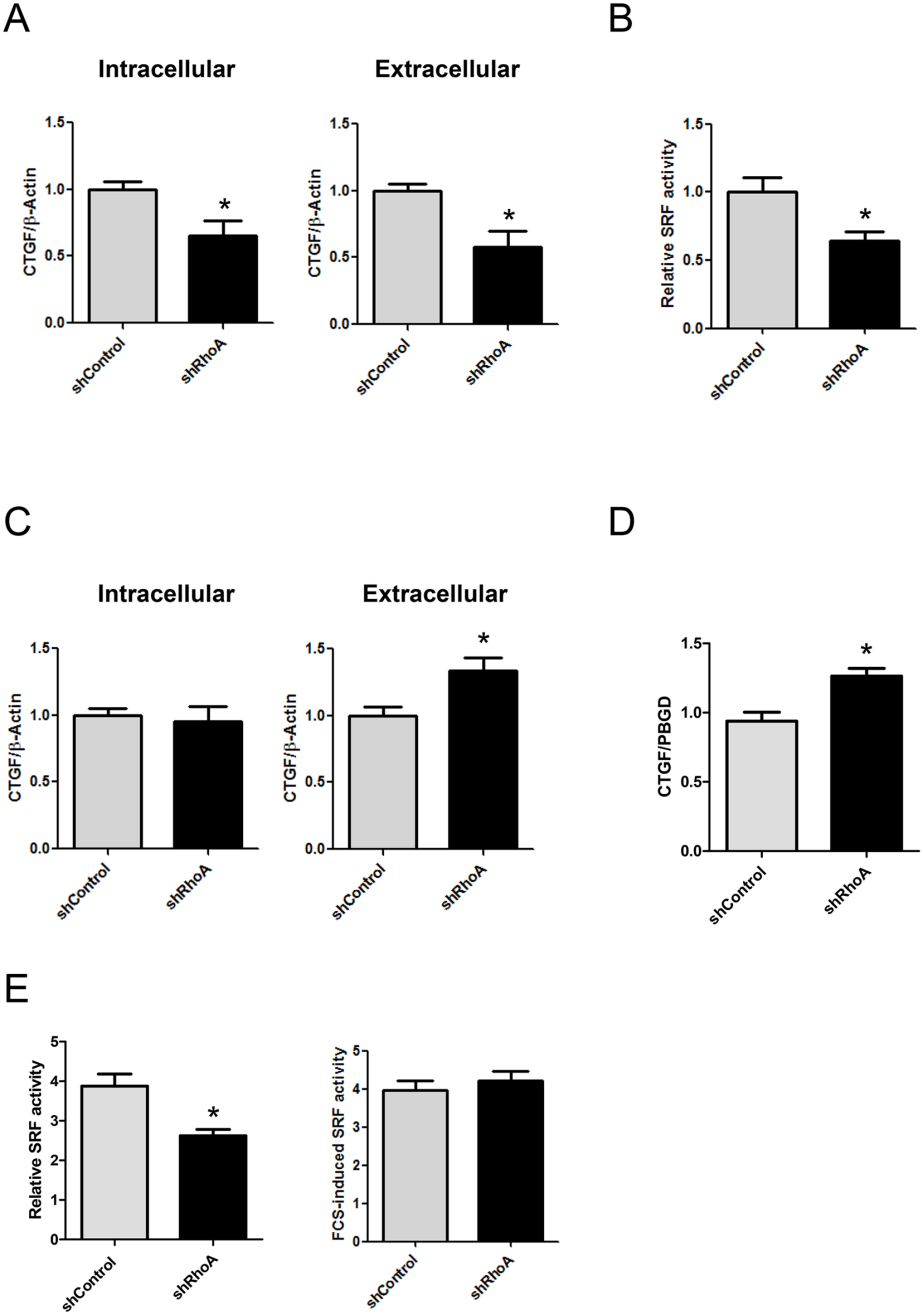
Expression and secretion of CTGF is impaired with reduced RhoA expression in low serum conditions. A) Bar graph summary of intracellular (left) and extracellular (right) CTGF under serum reduced conditions (1%) (means ± SEM, n = 6–7, *p < 0.05). Whole cell lysates and culture media were obtained from shControl and shRhoA NRCF and the relative changes of CTGF normalized to β-actin in shRhoA NRCF to shControl are given. B) Luciferase assay depicting the relative serum response factor activation in low serum conditions (1%). The relative change of shRhoA to shControl NRCF is given (means ± SEM, n = 5, *p < 0.05). C) Bar graph summary of intracellular (left) and extracellular (right) CTGF under high serum conditions (10%) (means ± SEM, n = 6–7, *p < 0.05). The relative changes of CTGF normalized to β-actin in shRhoA NRCF to shControl are given. D) Bar graph summary of real-time PCR data for CTGF in shControl and shRhoA NRCF in high serum conditions (10%) normalized to PBGD and related to shControl (means ± SEM, n = 4 versus PBGD, *p < 0.05). E) Luciferase assay depicting the total serum response factor activation in high serum conditions (10%) (left) and the total serum response factor activity in high serum conditions (10%) (right) of shControl and shRhoA NRCF (means ± SEM, n = 5).

Myofibroblasts display an altered secretory behavior. One of the factors increased in fibrotic tissue but whose function is still under investigation is the connective tissue growth factor (CTGF). In low-serum conditions, the intracellular and extracellular CTGF was found to be reduced in shRhoA NRCF (Fig 6A). This was accompanied by a decrease in the activity of the serum response factor (SRF) (Fig 6B), which was demonstrated before to regulate CTGF gene transcription in a actin-dependent manner by direct binding to the CTGF-promoter [20]. In contrast, in the presence of 10% serum, CTGF expression was increased in the conditioned medium of shRhoA NRCF (Fig 6C) and on mRNA level (Fig 6D). The serum-induced SRF activity was lower in shRhoA compared to shControl NRCF. However, the relative increase induced by serum was similar (Fig 6E) arguing for a RhoA-independent regulation of gene transcription in the presence of serum. To further validate this, we studied the transcription of other fibrosis-associated factors like TGF-β1, collagen 1a1 and 3a1 and biglycan in the presence of serum and found them all to be up-regulated in shRhoA-NRCF (S3 Fig).

### ROCK and HDAC6 differentially regulate cell morphology and proliferation

**Fig 7:**
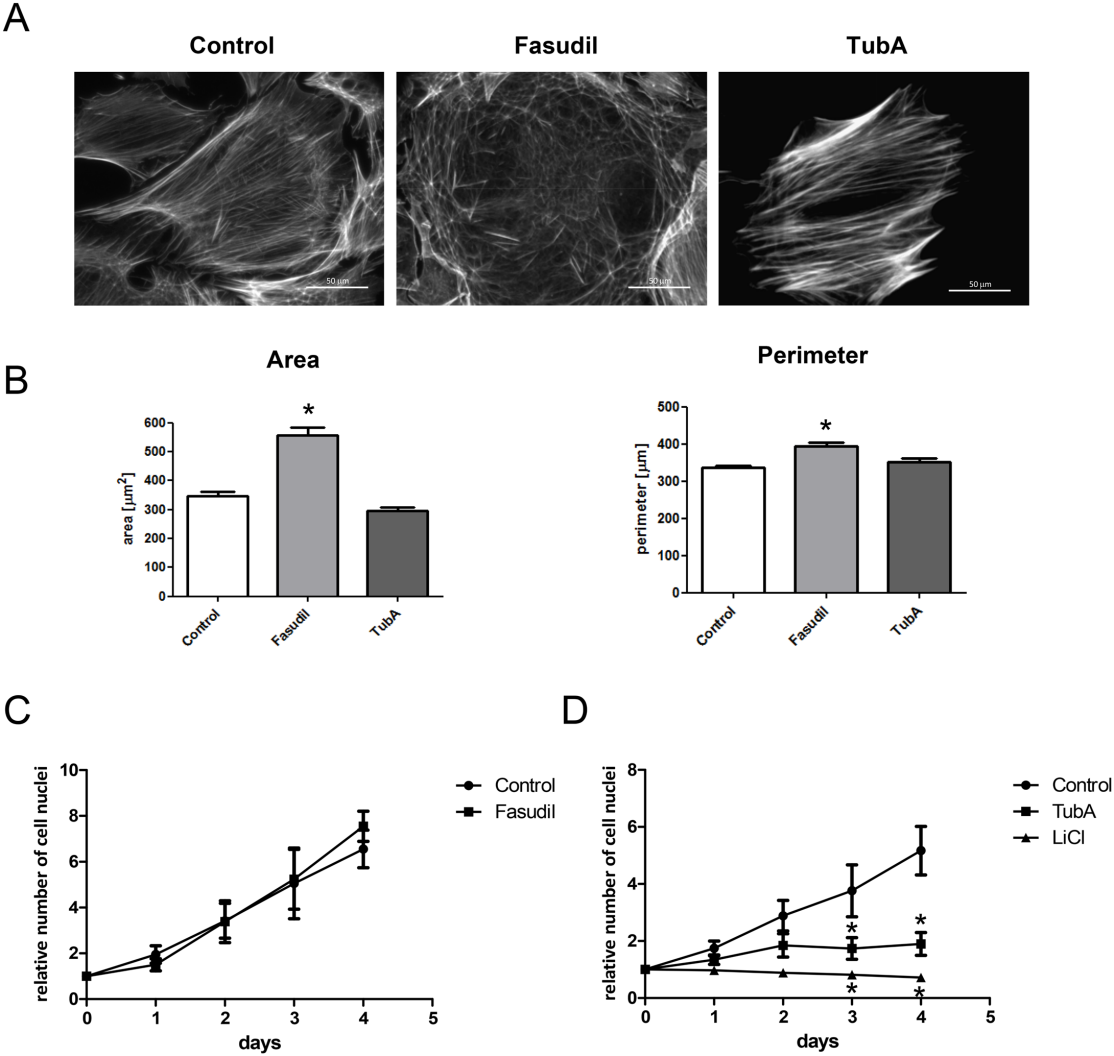
Actin disruption, cell morphology, migratory and proliferative performance of RhoA knockdown NRCF can be mimicked using ROCK and HDAC6 inhibitors. A) Representative actin stainings of control, fasudil (10 μM) and TubA (5 μM) treated NRCF (400x). B) Morphometric analysis of cell area (left) and perimeter (right) of control, fasudil (10 μM) and TubA (5 μM) treated NRCF (means ± SEM, n = 3, 50 NRCF per condition, *p < 0.05). C) Proliferation-DAPI assay of control and fasudil treated NRCF over a time course of 4 days (means ± SEM, n = 5, *p < 0.05). D) Proliferation-DAPI assay of control, TubA and LiCl (50 mM) treated NRCF over a time course of 4 days (means ± SEM, n = 3, *p < 0.05).

In order to identify the RhoA effectors involved in the observed changes in shRhoA NRCF, we performed experiments with wild type fibroblasts and inhibitors including the ROCK inhibitor fasudil and the HDAC6 inhibitor tubastatin A (TubA). TubA treatment led to a 4-fold increase in tubulin acetylation whereas no change was observed when cells were treated with fasudil (S4 Fig). In the first step, we analyzed the effect of these inhibitors on the cell morphology and the cytoskeleton of NRCF. Fasudil impaired the formation of higher order actin structures similar to the RhoA knockdown and increased the area of attached cells by 2-fold. TubA had no effect on cell size and on the actin cytoskeleton (Fig 7A and 7B). With respect to cell proliferation, we show that fasudil did not impair cell proliferation thus a role of ROCK can be excluded (Fig 7C). In contrast, TubA completely suppressed the FCS-induced proliferation. To further link the importance of the tubulin acetylation status to the proliferative capacity of NRCF we treated the cells with LiCl which was demonstrated to mobilize the α-Tubulin acetyltransferase 1 (α-TAT1) and moderately increase tubulin acetylation [21]. In NRCF LiCl increased tubulin acetylation 2.2-fold (S4 Fig) and fully inhibited cell proliferation, thereby emphasizing the role of this cytoskeletal modification in mitogenic processes. The observed effects, like the change in α-sm-actin expression and in the Golgi apparatus morphology were not affected by fasudil or TubA (S4 Fig).

### NRCF migration involves ROCK and mDia1

**Fig 8:**
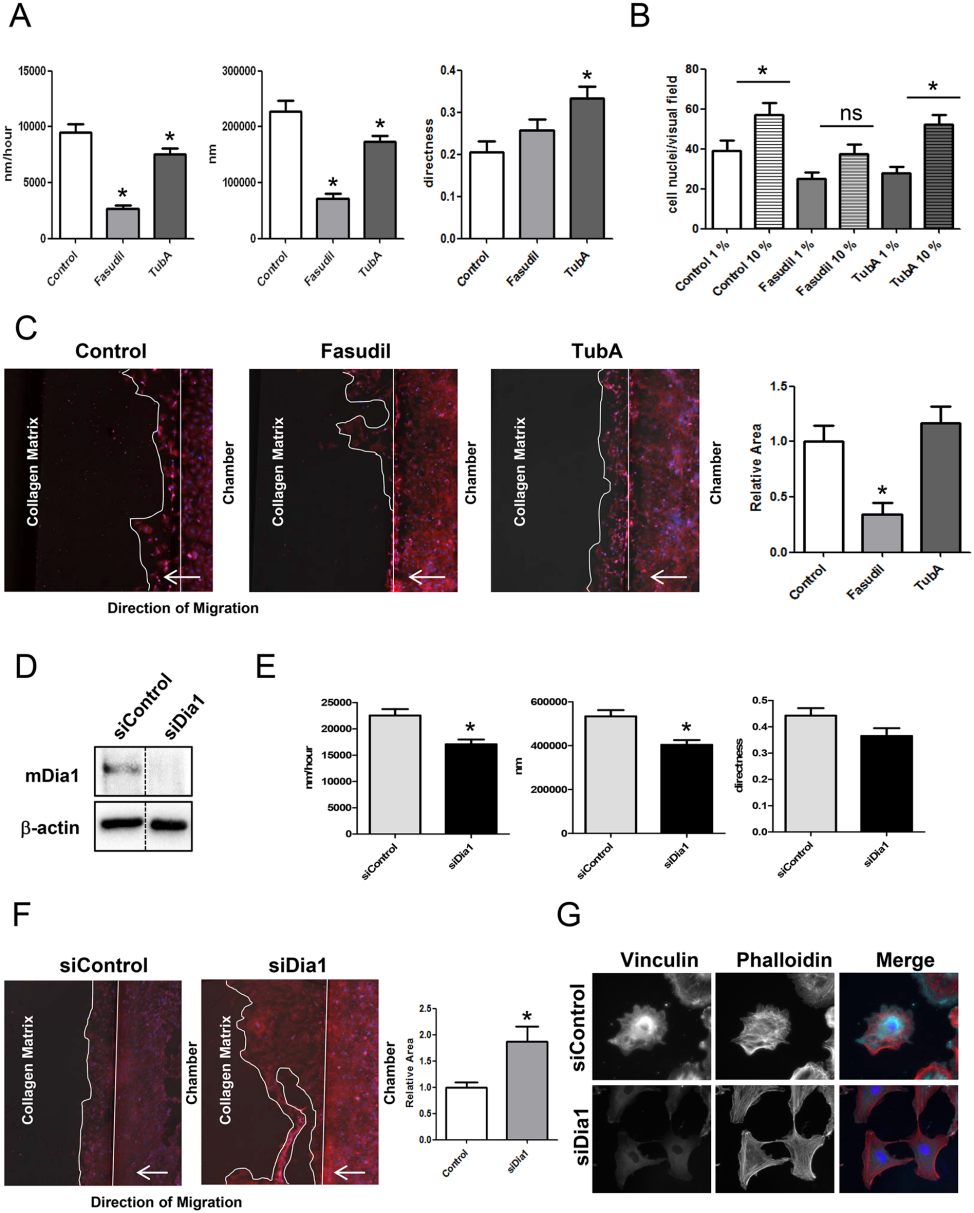
Migration through a collagen matrix is impaired after ROCK inhibition but improved after mDia1 knockdown. A) Bar graph summary of average migration velocity (left), absolute distance (middle) and directness (right) of control, fasudil and TubA treated NRCF migrating on a plane cell culture surface (means ± SEM, n = 3, 15 NRCF per condition, *p < 0.05). B) Bar graph summary of cell migration of control, fasudil and TubA treated NRCF through a porous membrane (pore size: 8 μm) in the presence of low serum (1%) and high serum (10%) (means ± SEM, n = 4, *p < 0.05). C) Representative images of control, fasudil and TubA treated NRCF migrating through a collagen membrane in ibidi μ-slides^3D^. The cells were stained for total actin and with DAPI (left). Bar graph summary of cell migration of control, fasudil and TubA treated NRCF through a collagen matrix (1,5% in DMEM) in ibidi μ-slides^3D^. As a bait FCS (10%) was used (means ± SEM, n = 8, *p < 0.05) (right). D) Representative immunoblot of mDia1 and β-actin in whole cell lysates from shControl and siDia1 NRCF (left). E) Bar graph summary of average migration velocity (left), absolute distance (middle) and directness (right) of siControl and siDia1 NRCF migrating on a plane cell culture surface (means ± SEM, n = 3, 15 NRCF per condition, *p < 0.05). F) Representative images of siControl and siDia1 NRCF migrating through a collagen membrane in ibidi μ-slides^3D^. The cells were stained for total actin and with DAPI (left). Bar graph summary of cell migration of siControl and siDia1 NRCF through a collagen matrix (1,5% in DMEM) in ibidi μ-slides^3D^. As a bait FCS (10%) was used (means ± SEM, n = 7, *p < 0.05) (middle). G) Immunofluorescence staining of vinculin (green), actin (red) and DAPI (blue) (320x) of siControl and siDia1 NRCF inside a collagen matrix (1,5% in DMEM) (right).

To investigate the effect of RhoA on cell migration in the different assay systems, we first studied the effect of ROCK and HDAC6 inhibition on the ability of WT NRCF to migrate on a plane surface. Treatment with fasudil inhibited migration velocity and absolute migration distance similar to the knockdown of RhoA (Fig 8A). In contrast, in the transwell assay and in the *in matrix* migration assay, treatment with fasudil did not increase migration as shown for shRhoA NRCF but significantly suppressed it (Fig 8B and 8C). HDAC6 inhibition by TubA showed only moderate effects on planar cell migration and had no effect in the other assays (Fig 8A–8C). As ROCK inhibition did not resemble the knockdown of RhoA, we further downregulated the RhoA effector Dia1 by siRNA transfection (Fig 8D) and studied planar cell migration. The knockdown of Dia1 significantly reduced migration velocity and distance (Fig 8E), however, not as effectively as the knockdown of RhoA or inhibition of ROCK. In contrast, in the collagen matrix migration assay repression of Dia1 expression resulted in an increase in migration capacity (Fig 8E). The complexity of the actin cytoskeleton in matrix-embedded siDia1-NRCF was reduced (Fig 8F).

### Impact of a RhoA knockdown on the viscoelastic and contractile parameters of 3D engineered tissues

As the specific genetic knockout of proteins in cardiac fibroblasts is not possible to date, we analyzed the outcome of a knockdown of RhoA in cardiac fibroblasts in homogenous and heterogeneous 3D engineered tissue models.

**Fig 9:**
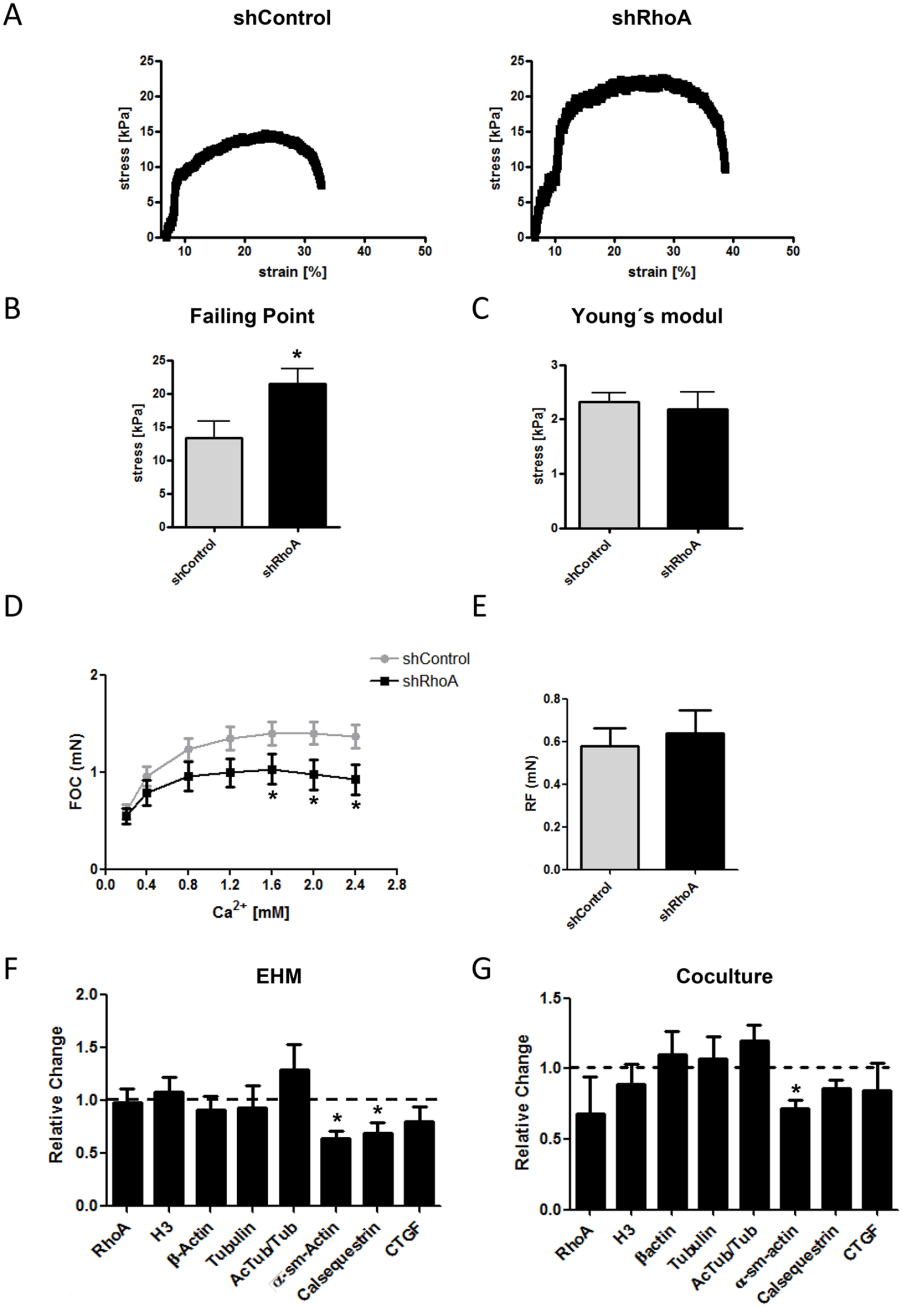
Impact of the RhoA knockdown on viscoelastic and contractile properties of engineered tissues. A) Representative stress-strain curves of ECT composed of shRhoA and shControl NRCF. B) Evaluation of the Failing Point (n = 6) and C) the Young´s modulus (n = 4–5) (means ± SEM, *p < 0.05). D) Evaluation of the force of contraction (FOC) in EHM supplemented with either shRhoA or shControl NRCF in the presence of different calcium concentrations (means ± SEM, n = 3, 2–4 EHM per condition and experiment, *p < 0.05) E) Given is the mean resting force of all EHM at 2 mM calcium as determined by the Frank-Starling mechanism (means ± SEM, n = 3, 2–4 EHM per condition and experiment). F) Quantification of an immunoblot analysis of cytoskeletal proteins, calsequestrin, intracellular CTGF and H3 in cell lysates obtained from shControl and shRhoA NRCF complemented EHM. The values are normalized to vimentin and given relative to the expression in shControl NRCF containing EHM (means ± SEM, n = 3, 2–4 EHM per condition were pooled and analyzed, *p < 0.05). G) Quantification of an immunoblot analysis of cytoskeletal proteins, calsequestrin, intracellular CTGF and H3 in cell lysates obtained from shControl and shRhoA NRCF co-cultured with neonatal rat cardiomyocytes for 7 days. The values are normalized to vimentin and given relative to the expression in shControl NRCF co-cultures (means ± SEM, n = 6, *p < 0.05).

In the first step, we used shRhoA and shControl NRCF and prepared engineered connective tissues (ECT). The viscoelastic properties of the ECT were determined by rheometric destructive tensile strength measurements (Fig 9A). For ECT prepared from shRhoA cells significantly more ultimate stress was needed until rupture of the tissue (failure point) (Fig 9B). However, ECT prepared from shControl and shRhoA NRCF showed no difference in the Young´s modulus, hence, in the stiffness of the tissue (Fig 9C).

In the next step, we added lentiviral transduced cardiac fibroblasts to a mixture of freshly isolated cardiac cells composed of cardiomyocytes and native fibroblasts, and prepared engineered heart muscle (EHM). Following 7 days condensation phase and 7 days stretching we analyzed the contractile parameters of the EHM in an organ bath. Interestingly we found that EHM containing additional shRhoA NRCF showed a significantly reduced force of contraction (FOC) in the presence of calcium concentrations higher than 2mM (Fig 9D). No impact on the diastolic resting force (RF) could be detected (Fig 9E). Biochemical analyses of the protein composition revealed a significant reduction in α-sm-actin in shRhoA complemented EHM as well as a significant decrease in the cardiomyocyte marker calsequestrin (Fig 9F). Similar findings for α-sm-actin, calsequestrin and CTGF expression could be observed in a co-culture experiment of neonatal rat cardiomyocytes with tranduced NRCF (Fig 9G).

## Discussion

In this study we demonstrate that the monomeric GTPase RhoA regulates the actin cytoskeleton, the structure of the Golgi apparatus, gene expression, proliferation and migration in cultured cardiac fibroblasts. Moreover, we show that RhoA regulates the viscoelastic properties of ECT and the contractile function of EHM.

In general, our findings support the idea of RhoA as a “myofibroblastic” protein as suggested before. With respect to gene transcription it was shown that RhoA positively regulates the expression of the myofibroblast marker α-sm-actin in response to tensile force [22] and angiotensin II [23] as well as collagen VIII expression [24]. The underlying signaling was demonstrated to involve the actin-dependent regulation of the SRF by its co-factor [22].

Our data supports the role of RhoA/SRF signaling in the regulation of α-sm-actin in cultured cardiac fibroblasts. However, in the transcriptional regulation of other fibrosis-associated genes the function of RhoA seems to be stimulus dependent. In isolated cardiac fibroblasts and other cell types it was demonstrated that RhoA induces the up-regulation of CTGF, TGF-beta and collagen in response to angiotensin II [23, 25]. In contrast, our data demonstrates that in the presence of serum a reduction in RhoA results in an up-regulation of CTGF, TGF-beta, Col1a1, Col3a1 and the collagen organizer biglycan. This might be due to an alternative regulation of gene transcription in shRhoA NRCF by other co-factors than MRTF and/or other transcription factors than SRF. In a study on direct SRF target genes neither TGF-beta1, Col1a1, Col3a1 nor biglycan were identified, only CTGF was proposed and shown before to be a MRTF/SRF target gene [26] [20]. Besides, CTGF is not an exclusive target of MRTF/SRF as it can also be induced by RhoA-independent ETS–1/SRF-driven gene transcription [27]. In line with this, we show that under low serum conditions, when SRF is dependent on the actin status and thus on RhoA, SRF activity as well as CTGF expression was reduced in shRhoA NRCF. In high serum conditions, CTGF expression was increased in shRhoA NRCF. However, the high serum led to a comparable activation of SRF in shControl and shRhoA NRCF, arguing for a RhoA independent mechanism. So far, it is not clear, which other signaling pathways might play a role in the observed SRF induction. However, CTGF is known to be regulated by diverse other signal cascades including the Hippo pathway and TGF-beta signaling [28, 29] How the other genes are up-regulated is also not clear, but it is likely that the increase in Col1a1, Col3a1 and biglycan is a consequence of TGF-beta-dependent gene regulation [30, 31].

The role of RhoA in cardiac fibroblast proliferation is less established than its function in gene transcription. It was shown that simvastatin inhibits the FCS-induced proliferation of human atrial myofibroblasts. Based on the reversibility of this effect by geranylgeranyl-pyrophosphate and changes in RhoA localization it was postulated that RhoA drives FCS-induced proliferation This involves ROCK signaling as demonstrated by the use of a ROCK inhibitor [32]. ROCK inhibition was also shown to inhibit the Angiotensin II-dependent proliferation of neonatal rat cardiac fibroblasts [33].

Our study confirms the role of RhoA in cardiac fibroblast proliferation. However, we could not observe an effect of the ROCK inhibitors fasudil and H1152p in serum-driven proliferation (data not shown). In our hands, mimicking the increase in tubulin acetylation mirrored the anti-proliferative effect observed in shRhoA NRCF. Up-regulation of tubulin acetylation by the αTat1 modifier LiCl or the HDAC6 inhibitor TubA led to a pronounced inhibition of NRCF proliferation. Although this has not been shown before for cardiac fibroblasts, there is other data supporting the role of acetylated tubulin in cell proliferation. For cancer cells it was demonstrated that increased tubulin acetylation by HDAC6 inhibition suppressed proliferation [34, 35]. Vice versa, in embryonic fibroblasts a loss of tubulin acetylation due to a genetic loss of αTat1 resulted in a strongly increased proliferation [36]. So far, it is unclear which of the tubulin-acetylation regulators are dysregulated in shRhoA NRCF. Besides αTat1 and HDAC6, other tubulin deacetylases like sirtuins must be considered as sirtuin inhibition was also shown to be anti-proliferative [37].

There are only a few publications analyzing cardiac fibroblast migratory behavior and the involvement of RhoA and ROCK. No data is available on the role of Dia1 in these cells. In line with other reports, we found that RhoA is important for the migration on a plane surface [23, 38]. In our study the repression of RhoA expression and the inhibition of ROCK decreased migration velocity and the absolute migrated distance. Moreover, we demonstrated that the knockdown of Dia1 also reduces migration ability in the same assay, however, the effect was less pronounced compared to RhoA knockdown or ROCK inhibition. In contrast, in the transwell and *in matrix* migration assay a higher migratory capacity of shRhoA NRCF was detected. This was the opposite for WT NRCF in the presence of fasudil, but a knockdown of Dia1 NRCF had a similar augmenting effect on the migration in a collagen matrix. These findings are quite unexpected and have not been described before. In general, fibroblasts can migrate in different modes depending on the dimensionality and consistency of the substratum. The two corners of migration are the lamellipodia-dependent, “mesenchymal” migration and the blebbing-dependent amoeboid migration. In 2D cultures, fibroblasts usually migrate lamellipodia-dependent which requires a bipolar cell morphology. As repression of RhoA signaling in NRCF led to a loss in cell polarity it can be assumed that this partly explains the observed phenotype in the 2D model. Moreover, RhoA/ROCK activity is also important for tail retraction during migration. The blebbing-dependent amoeboid migration was described to be typical for germ cells and tumor cells [39], but not for fibroblasts. However, fibroblasts can migrate in an intermixed way recently described as lobopodia-dependent migration. It was postulated that whether fibroblasts migrate lobopodia- or lamellipodia-dependent in 3D is dependent on the matrix itself and on the activity of RhoA [40, 41]. In RhoA knockdown NRCF more lamellipodia appear to be present than in the shControl cells. Whether this explains the higher speed in migration has to be elucidated further. Moreover, the differential role of the RhoA downstream effectors ROCK and Dia1 in NRCF migratory behavior needs further investigation. Another point that must be considered in the scenario of 3D migration of cardiac fibroblasts is the morphology of the cytoskeleton. In 2D, the actin cytoskeleton of around 25% of shControl NRCF is partly or purely geodesic, whereas in shRhoA cells the percentage is significantly lower. Interestingly, all control NRCF, independent whether they were tranduced, tranfected or untreated, keep these dome-structures in the collagen matrix. So far, nothing is known about the dynamics of these structures in a matrix, but it can be assumed that the dome structure is sterically hindering the migration through a meshwork. NRCF with a reduced expression of RhoA or Dia1 displayed fewer of these structures in the matrix and thus migration of those cells might be facilitated.

The data obtained in 2D cultures points to an ambivalent role of RhoA in the disease-associated phenotypic shift of cardiac fibroblasts to myofibroblasts. Therefore, we studied its role in 3D models. It is important to mention that all observed changes in ECT and especially in the EHM model are due to changes in cardiac fibroblasts and thus rely on auto-and paracrine signals. In ECT we found that the stiffness of the tissues, either composed of shControl or shRhoA NRCF, are comparable, as demonstrated by the Young’s modulus. However, the ultimate stress, which is necessary to reach the failure point was significantly higher in shRhoA-ECT. At this point, it is difficult to explain this satisfactorily. ECT are composed of a cell and a matrix component both playing a role in the viscoelastic properties. On the one hand, shRhoA NRCF display alterations in focal adhesion sites and in adhesion itself. Therefore, they most likely show differences in cell-matrix binding, also in a tissue. Moreover, the content of α-sm-actin is reduced which could impair the intracellular response to mechanic stretch. On the other hand, in the presence of serum we demonstrated an increase in the expression of collagens and biglycan probably stabilizing the ECM. Collagens might mainly increase the stiffness of tissues, but for biglycan it was demonstrated that its loss can cause spontaneous aortic dissection and rupture emphasizing its role in tissue toughness and integrity [42].

Finally, in the EHM model we found that tissues containing a certain proportion of cardiac fibroblasts with reduced RhoA expression are less contractile than the respective controls. This can only be explained by a paracrine effect. The reduced expression of calsequestrin points to the regulation of cardiomyocyte survival. The role of RhoA and ROCK in cardiomyocyte cell survival is well established in mouse models [43]. However, the contribution of cardiac fibroblasts is not clear as no fibroblast-specific genetic models are published so far for the respective proteins. In the same model, there is only indirect data supporting our findings by a study published by Mühlhäuser et al. describing that EHM treated with atorvastatin decreased contractile force up to 50% dependent on the concentration used [44].

## Conclusion

**Fig 10:**
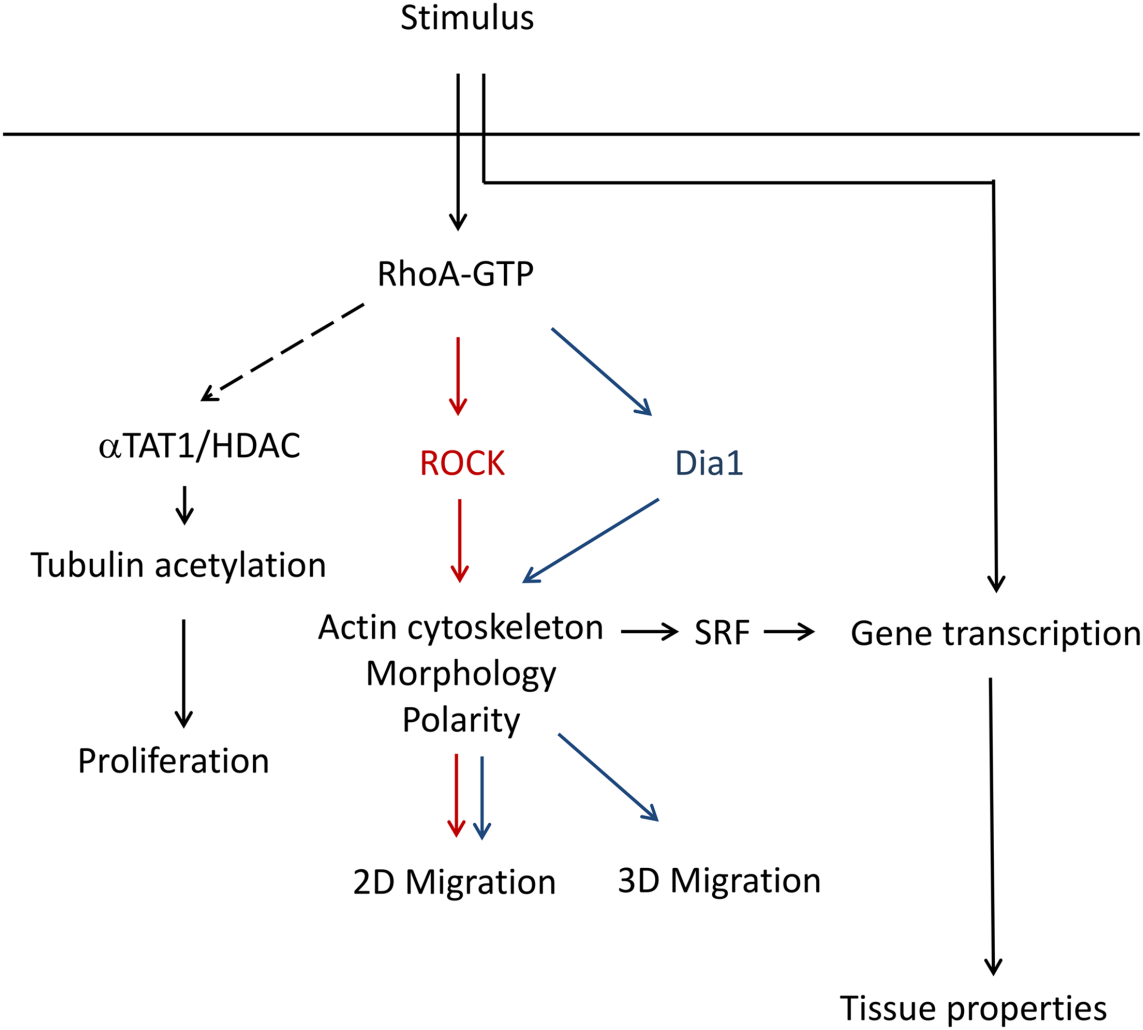
Summary scheme of proposed mechanism.

The data obtained in this study argues for an ambivalent role of RhoA in the regulation of cardiac myofibroblast characteristics. Our study introduces RhoA as a balancing mediator of fibroblast activation and senescence. By shifting the influence of its downstream effectors the monomeric GTPase can promote extracellular matrix deposition and an improved 3D migration as well as minimize proliferation, change cytoskeleton composition and reduce cell polarity (Fig 10). How RhoA in cardiac fibroblasts is capable to influence cardiomyocyte survival via endo- or paracrine signaling is still under investigation and has to be addressed in further studies.

## Supporting Information

S1 Fig A) RhoA activity assay of shControl and shRhoA NRCF using angiotensin II (100 nM) for 90 s as a stimulus. Shown is the immunoblot analysis of RhoA-GTP determined by pulldown assay, total RhoA and tubulin in total cell lysates. B) Bar graph summary of β-actin protein expression normalized to total cell lysate obtained from shControl and shRhoA NRCF (means ± SEM, n = 6). Bar graph summary of real-time PCR data for RhoB. mRNA obtained from shControl and shRhoA NRCF was used. The values are normalized to PBGD and given relative to shControl (means ± SEM, n = 6) (left). C) Representative immunoblot of RhoC and β-actin (middle) and bar graph summary of RhoC protein expression normalized to β-actin (means ± SEM, n = 4) (right). Whole cell lysates were obtained from shControl and shRhoA NRCF. The relative change of shRhoA to shControl is given. D) Bar graph summary of real-time PCR data of HDAC6 normalized to PBGD and protein expression normalized to β-actin for HDAC6 in shControl and shRhoA NRCF related to shControl (means ± SEM, n = 7). E) Representative immunoblots of lysine acetylation pattern in whole cell lysates and particulates obtained from shControl and shRhoA NRCF is shown. On the left side of each condition the ponceauS staining is shown on the right side the corresponding immunoblot using an anti-acetyl-lysine antibody. The arrow marks the band of acetylated tubulin.
(TIF)

S2 Fig A) Bar graph summary of real-time PCR data for TGFβ, Col1A, Col3a and biglycan in shControl and shRhoA NRCF normalized to PBGD and related to shControl (means ± SEM, n = 7 versus PBGD, *p < 0.05).
(TIF)

S3 Fig A) Immunoblot analysis of acetylated tubulin normalized to β-actin in inhibitor treated wild type fibroblasts. Whole cell lysates were obtained from control, fasudil (10 μM), LiCl (50 mM) and TubA (5 μg/mL) treated NRCF (means ± SEM, n = 6, *p < 0.05). B) Immunoblot analysis α-sm-actin normalized to β-actin in inhibitor treated wild type fibroblasts. Whole cell lysates were obtained from control, fasudil (10 μM) and TubA (5 μg/mL) treated NRCF (means ± SEM, n = 6). C) Structural analysis of Golgi apparatus size (left) and density (right) of control, fasudil (10 μM) and TubA (5 μg/mL) treated wild type NRCF as assessed by WGA-staining and fluorescence microscopy (means ± SEM, n = 3, 10 Golgi apparatus per condition).
(TIF)

S4 Fig Antibody list (I). (TIF)

S5 Fig Antibody list (II). (TIF)

S1 Video Life cell imaging over 24 h of shControl cells infected with LifeAkt-GFP adenovirus. (ZIP)

S2 Video Life cell imaging over 24 h of shRhoA cells infected with LifeAkt-GFP adenovirus. (ZIP)

S3 Video Life cell imaging over 24 h of siControl NRCF. (ZIP)

S4 Video Life cell imaging over 24 h of siDia1 NRCF. (ZIP)
