# Supplementary figures and images for: RhoA Ambivalently Controls Prominent Myofibroblast Characteritics by Involving Distinct Signaling Routes
Source: PLoS One. 2015 Oct 8;10(10):e0137519. doi: 10.1371/journal.pone.0137519 (PMC4598021; doi:10.1371/journal.pone.0137519)

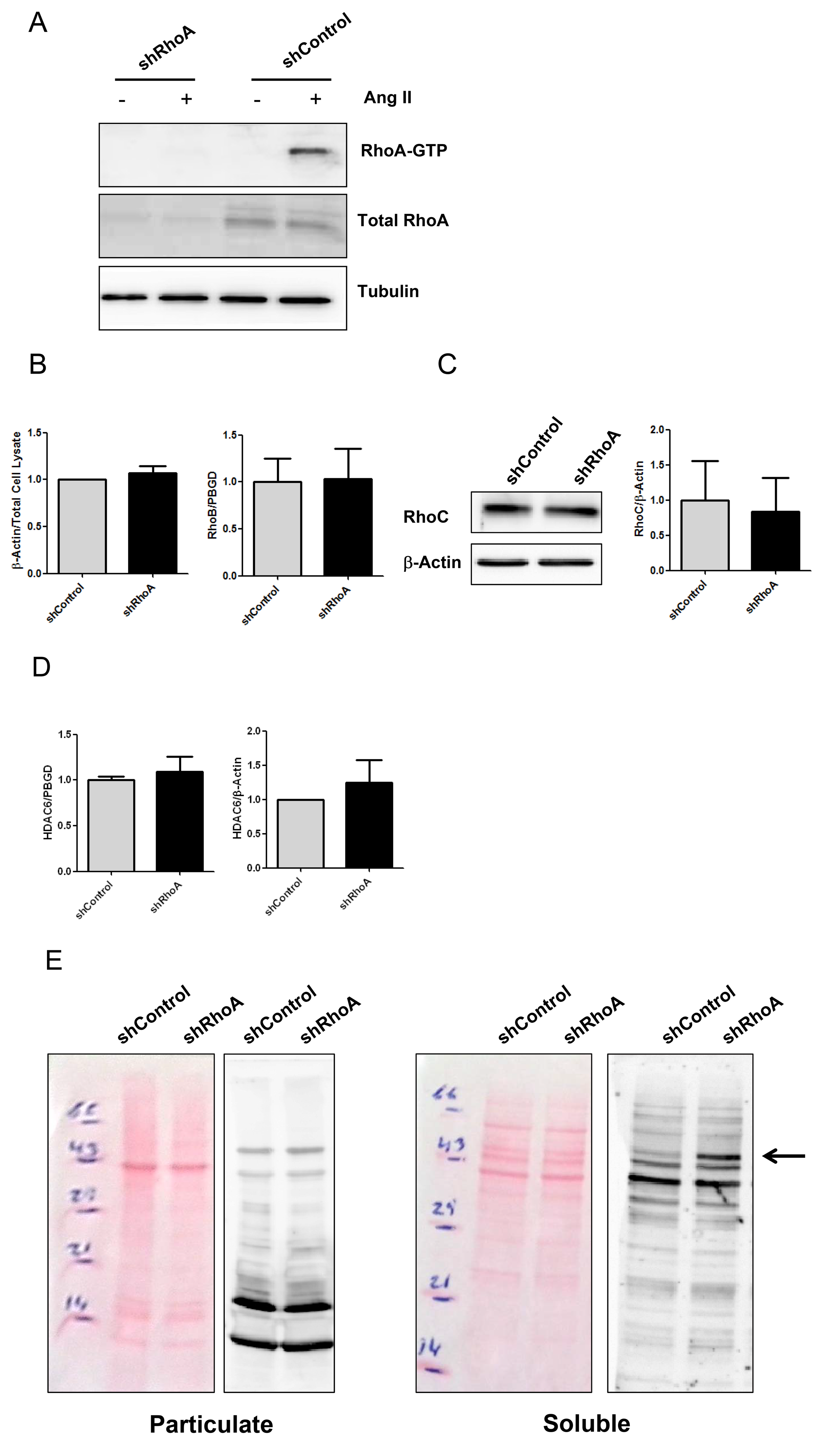

Supplement: S1 Fig — A) RhoA activity assay of shControl and shRhoA NRCF using angiotensin II (100 nM) for 90 s as a stimulus. Shown is the immunoblot analysis of RhoA-GTP determined by pulldown assay, total RhoA and tubulin in total cell lysates. B) Bar graph summary of β-actin protein expression normalized to total cell lysate obtained from shControl and shRhoA NRCF (means ± SEM, n = 6). Bar graph summary of real-time PCR data for RhoB. mRNA obtained from shControl and shRhoA NRCF was used. The values are normalized to PBGD and given relative to shControl (means ± SEM, n = 6) (left). C) Representative immunoblot of RhoC and β-actin (middle) and bar graph summary of RhoC protein expression normalized to β-actin (means ± SEM, n = 4) (right). Whole cell lysates were obtained from shControl and shRhoA NRCF. The relative change of shRhoA to shControl is given. D) Bar graph summary of real-time PCR data of HDAC6 normalized to PBGD and protein expression normalized to β-actin for HDAC6 in shControl and shRhoA NRCF related to shControl (means ± SEM, n = 7). E) Representative immunoblots of lysine acetylation pattern in whole cell lysates and particulates obtained from shControl and shRhoA NRCF is shown. On the left side of each condition the ponceauS staining is shown on the right side the corresponding immunoblot using an anti-acetyl-lysine antibody. The arrow marks the band of acetylated tubulin. (TIF) [file pone.0137519.s001.tif]

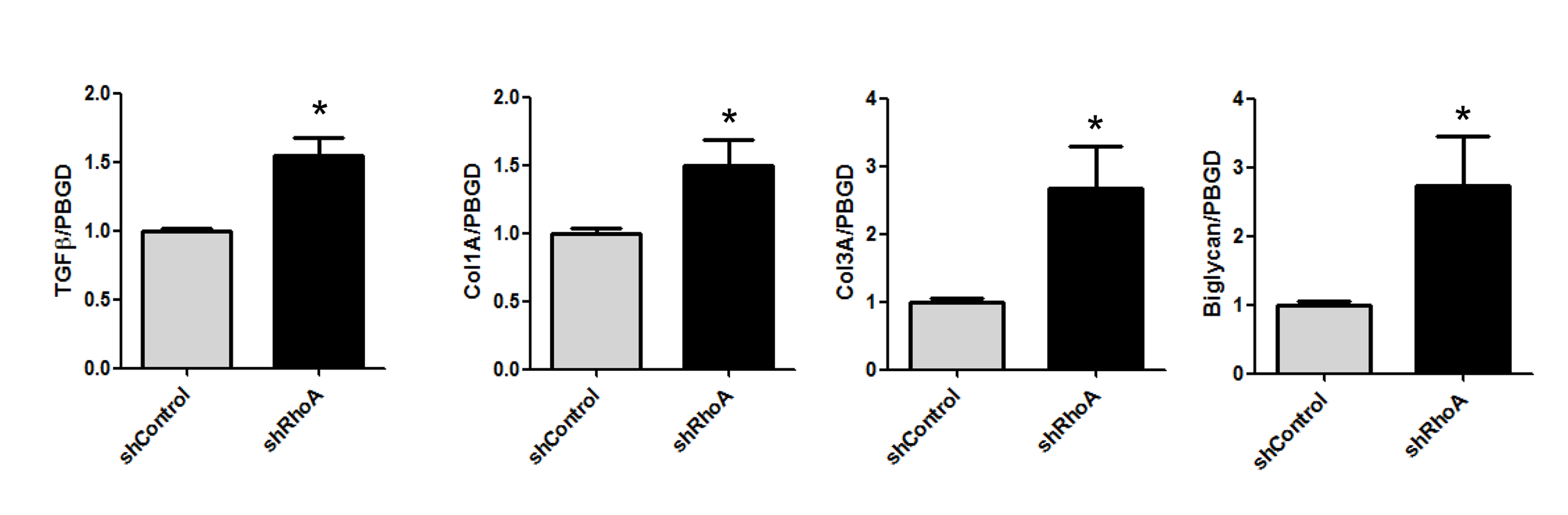

Supplement: S2 Fig — A) Bar graph summary of real-time PCR data for TGFβ, Col1A, Col3a and biglycan in shControl and shRhoA NRCF normalized to PBGD and related to shControl (means ± SEM, n = 7 versus PBGD, *p < 0.05). (TIF) [file pone.0137519.s002.tif]

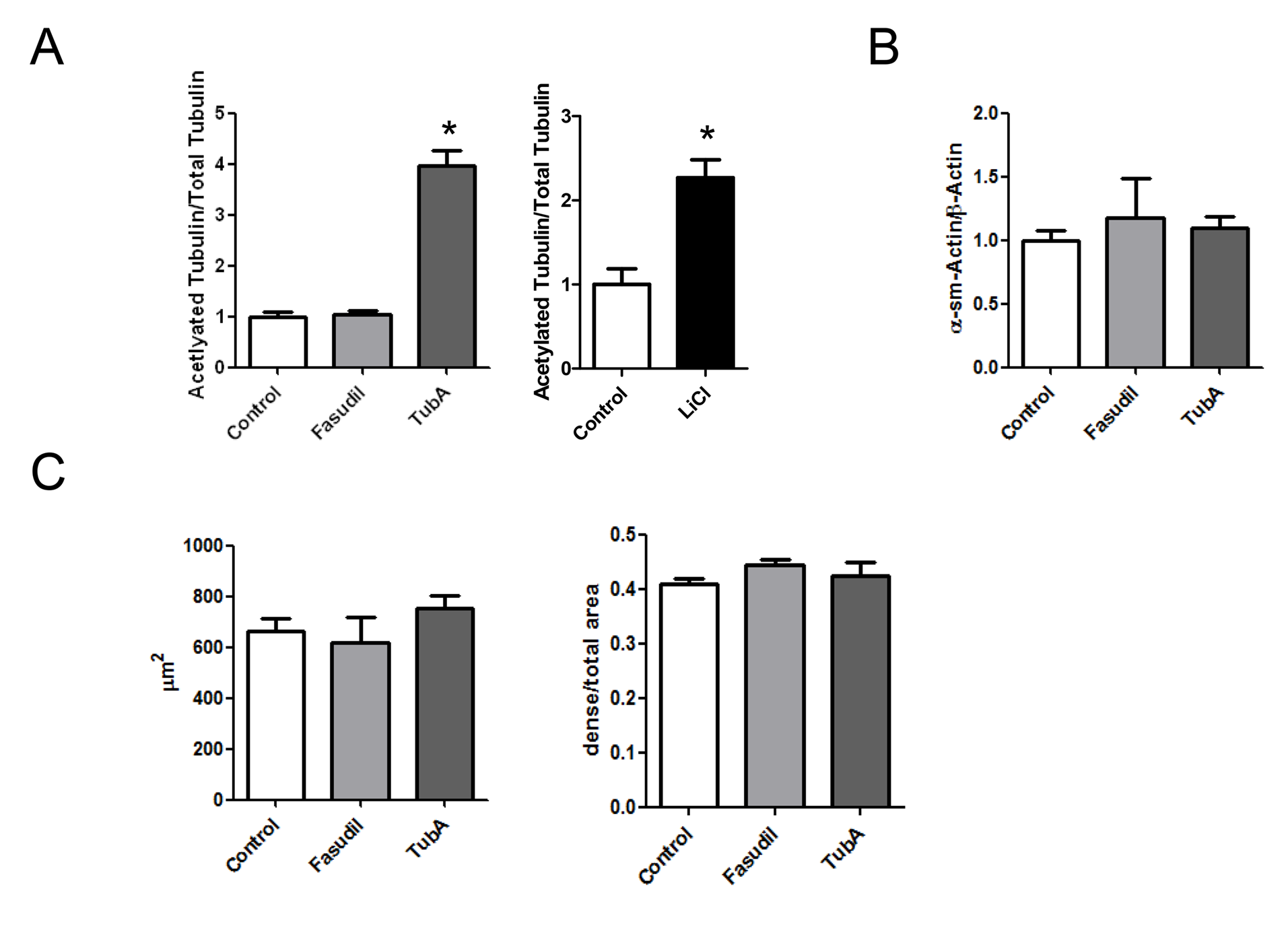

Supplement: S3 Fig — A) Immunoblot analysis of acetylated tubulin normalized to β-actin in inhibitor treated wild type fibroblasts. Whole cell lysates were obtained from control, fasudil (10 μM), LiCl (50 mM) and TubA (5 μg/mL) treated NRCF (means ± SEM, n = 6, *p < 0.05). B) Immunoblot analysis α-sm-actin normalized to β-actin in inhibitor treated wild type fibroblasts. Whole cell lysates were obtained from control, fasudil (10 μM) and TubA (5 μg/mL) treated NRCF (means ± SEM, n = 6). C) Structural analysis of Golgi apparatus size (left) and density (right) of control, fasudil (10 μM) and TubA (5 μg/mL) treated wild type NRCF as assessed by WGA-staining and fluorescence microscopy (means ± SEM, n = 3, 10 Golgi apparatus per condition). (TIF) [file pone.0137519.s003.tif]

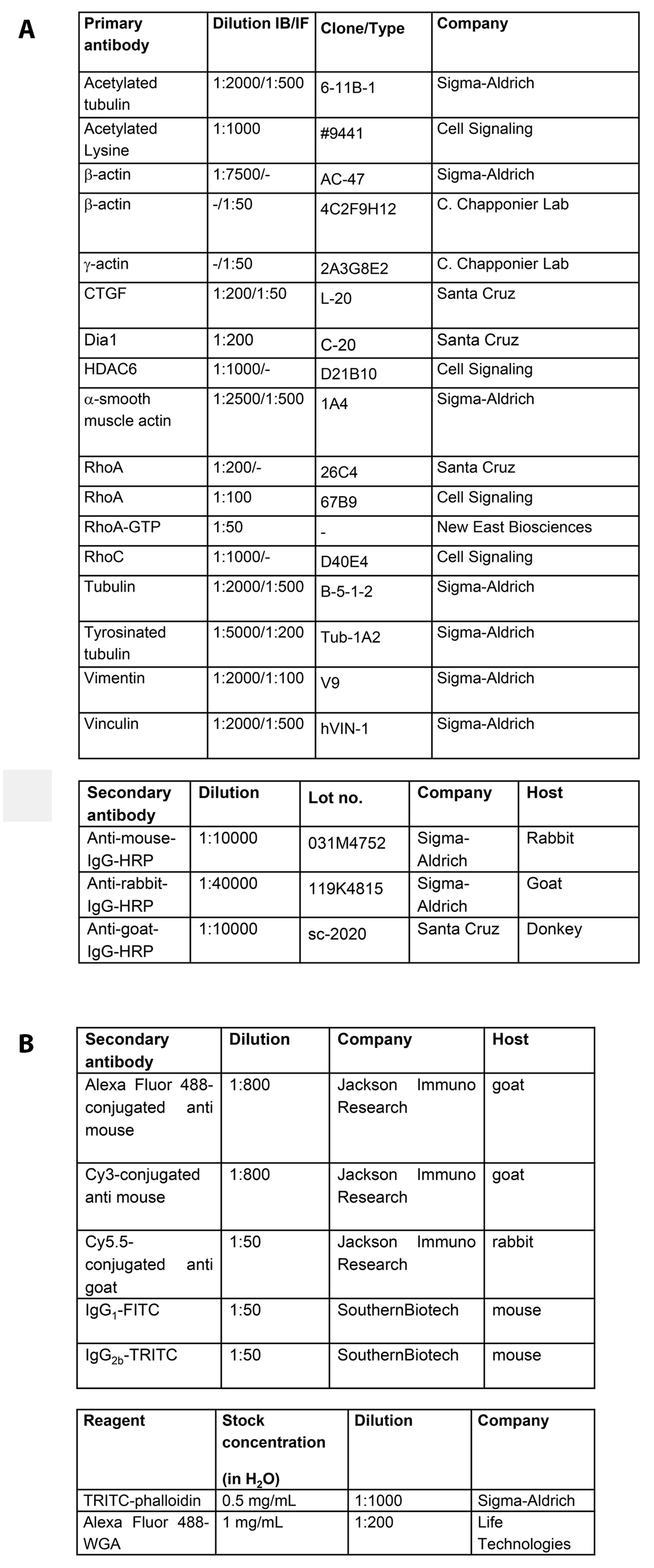

Supplement: S4 Fig — (TIF) [file pone.0137519.s004.tif]

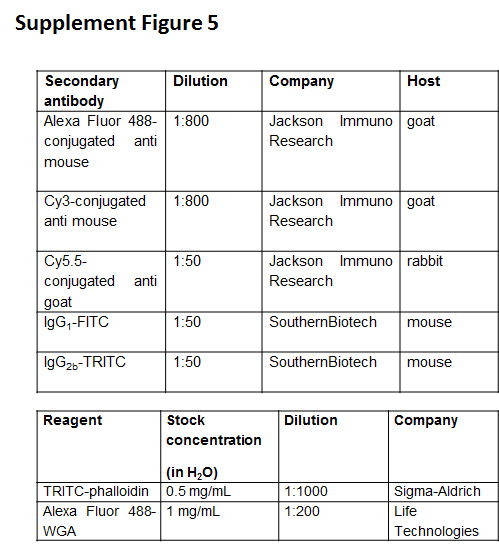

Supplement: S5 Fig — (TIF) [file pone.0137519.s005.tif]
